# Biodegradable and Excretable 2D W_1.33_C *i*‐MXene with Vacancy Ordering for Theory‐Oriented Cancer Nanotheranostics in Near‐Infrared Biowindow

**DOI:** 10.1002/advs.202101043

**Published:** 2021-10-29

**Authors:** Bangguo Zhou, Haohao Yin, Caihong Dong, Liping Sun, Wei Feng, Yinying Pu, Xiaoxia Han, Xiaolong Li, Dou Du, Huixiong Xu, Yu Chen

**Affiliations:** ^1^ Department of Medical Ultrasound Shanghai Tenth People's Hospital Ultrasound Research and Education Institute Tongji University Cancer Center Shanghai Engineering Research Center of Ultrasound Diagnosis and Treatment Tongji University School of Medicine Shanghai 200072 P. R. China; ^2^ Department of Ultrasound Zhongshan Hospital Fudan University and Shanghai Institute of Medical Imaging Shanghai 200032 P. R. China; ^3^ School of Life Sciences Shanghai University Shanghai 200444 P. R. China

**Keywords:** biodegradability, *i*‐MAX phase, *i*‐MXene, theranostics, vacancy ordering

## Abstract

MXenes, a new class of two‐dimensional (2D) nanomaterials, have shown enormous potential for biological applications. Notably, the development of 2D MXenes in nanomedicine is still in its infancy. Herein, a distinct W_1.33_C *i*‐MXene with multiple theranostic functionalities, fast biodegradation, and satisfactory biocompatibility is explored. By designing a parent bulk laminate in‐plane ordered (W_2/3_Y_1/3_)_2_AlC ceramic and optionally etching aluminum (Al) and yttrium (Y) elements, 2D W_1.33_C *i*‐MXene nanosheets with ordered divacancies are efficiently fabricated. Especially, theoretical simulations reveal that W_1.33_C *i*‐MXene possesses a strong predominance of near‐infrared (NIR) absorbance. The constructed ultrathin W_1.33_C nanosheets feature excellent photothermal‐conversion effectiveness (32.5% at NIR I and 49.3% at NIR II) with desirable biocompatibility and fast degradation in normal tissue rather than in tumor tissue. Importantly, the multimodal‐imaging properties and photothermal‐ablation performance of W_1.33_C‐BSA nanosheets are systematically revealed and demonstrated both in vitro and in vivo. The underlying mechanism and regulation factors for the W_1.33_C‐BSA nanosheets‐induced hyperthermia ablation are also revealed by transcriptome and proteome sequencing. This work offers a paradigm that *i*‐MXenes achieve the tailoring biomedical applications through composition and structure design on the atomic scale.

## Graphical abstract

A distinct two dimensional W_1.33_C *i*‐MXene with multiple theranostic functionalities, fast biodegradation, and satisfactory biocompatibilityhas been demonstrated. The underlying mechanism and regulation factors for the engineered W_1.33_C‐BSA nanosheetsinduced photothermal hyperthermia are revealed via genomics and proteomics. This work significantly expands the biomedical applications of *i*‐MXene‐based nanomedicines with the desirable biological effect and excellent theranostics performance.

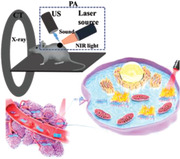

## 1 Introduction

Two‐dimensional (2D) nanomaterials with unique structural features and physicochemical properties have shown substantial potential for cancer theranostics.^[^ ^1^ ^]^ For example, graphene, transition‐metal dichalcogenides, monoelemental 2D materials and metal–organic frameworks. Since the Ti_3_C_2_ nanosheets first introduced in 2011, transition‐metal nitrides and carbonitrides (MXenes), as an emerging family of 2D materials with numbers of intriguing electronic and optical properties, have been explored for versatile applications in energy storage,^[^ ^2^ ^]^ electromagnetic interference shielding,^[^ ^3^ ^]^ environmental science,^[^ ^4^ ^]^ and biology.^[^ ^5^ ^]^ MXenes are generally synthesized by optionally etching the A group element from the matching layer‐structured MAX, in which “M” refers to early transition metal and “X” corresponds to carbon (C) and/or nitrogen (N). Owing to the attractive physicochemical properties, MXenes stand out among 2D materials and are expected as distinct state‐of‐the‐art platforms to promote the biomedical researches.^[^ ^5^, ^6^, ^7^ ^]^ For instance, the hydrophilic surface and low cytotoxicity highlight the biosafety and clinical translation potential of MXene‐based nanoplatforms. Furthermore, the broad and strong absorbance in near‐infrared (NIR) region as well as high light‐to‐heat conversion efficiency has rendered MXenes attractive for photoacoustic (PA) imaging and photothermal therapy (PTT). Based on abundant oxygen‐containing groups onto the surface, the nanoscale MXenes exhibit excellent surface‐engineering capability which can enhance the colloidal stability and prolong the in vivo blood circulation.^[^ ^5^, ^6^, ^8^ ^]^ However, up to now, even though more than 30 MXenes have been explored, only several types of MXenes including Ti_3_C_2_, Nb_2_C, Mo_2_C, V_2_C, and Ta_3_C_4_ have been used in biomedicine.^[^ ^9^ ^]^ Bio‐safety issues of these MXenes including biodegradation and excretion behaviors are considered as a crucial issue to be addressed. To the best of our knowledge, most types of MXenes are featured by high crystallinity and accompany with low defects, making them difficult to decompose in the physiological environment.^[^ ^10^ ^]^ It is highly necessary but challenging to explore new members of 2D MXenes with multiple theranostic functionalities, fast biodegradation, and satisfactory biocompatibility for guaranteeing their versatile biomedical applications and further clinical translations.

Owing to the high atomic number (*Z* = 74), strong X‐ray attenuating potency and intense absorbance in the NIR region, tungsten (W)‐based nanosystems have been explored as a kind of potential candidates for cancer theranostics.^[^ ^11^ ^]^ It follows that a particular potential application of W‐based 2D nanomaterial is biomedical field. The driving force to explore W‐based MXenes is goal‐oriented; however, it has been predicted that none of the ternary W‐based MAX phases are stable, which makes it difficult to synthesize W‐based MXene.^[^ ^12^, ^13^ ^]^ It is necessary to explore suitable fabrication strategies for W‐based MXenes. MAX phases containing different components and their corresponding MXenes could be obtained by structural designing at the atomic level. The reports show that out‐of‐plane and in‐plane chemical ordering, termed as *o*‐MAX and *i*‐MAX, have been explored in the MAX phase. The quaternary *i*‐MAX phase is characterized by in‐plane chemical order with two components M^1^ and M^2^ in a ratio of 2:1, following the general equation of (M^1^_2/3_M^2^_1/3_)_2_AC.^[^ ^13^, ^14^, ^15^, ^16^ ^]^ The selective removal of the A element, commonly Al, and M^2^ elements during etching results in M^1^_1.33_C MXene, called *i*‐MXene, with vacancies, in which the vacancies are ordered, endowing remarkable benefits.^[^ ^14^ ^]^ Owing to their unconventional structure and composition, together with advanced theoretical prediction and experimental measurements, it has promising applications in energy storage and conversion.^[^ ^16^, ^17^ ^]^ However, the application in other fields of *i*‐MXene is rarely reported, especially in biomedicine. Therefore, it is highly motivated to exploit appropriate strategies to synthesize W‐based *i*‐MXene and extend its biomedical application.

**Scheme 1:**
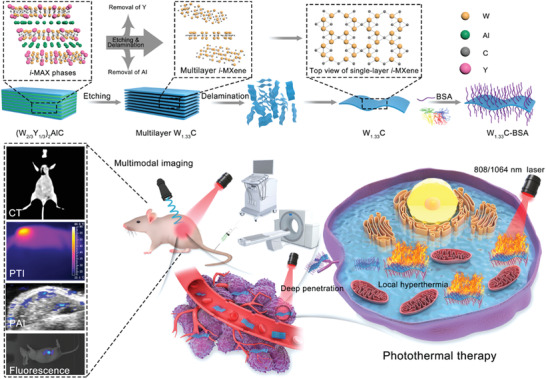
Schematic illustration on the construction of 2D W_1.33_C‐BSA nanosheets as a 2D phototherapeutic agent for multimodal‐imaging‐guided cancer treatment.

Herein, we report a W‐based 2D *i*‐MXene for multimodal‐imaging‐guided cancer theranostics (**Scheme** **1**). Quaternary *i*‐MAX phase, (W_2/3_Y_1/3_)_2_AlC with in‐plane chemical order, was fabricated through structural design on the atomic level. W_1.33_C‐based *i*‐MXenes could be produced by specifically remove both Y and Al elements from the corresponding *i*‐MAX phase bulks, which feature ordered basal‐plane vacancies. Given the inefficiency of current experimental trial‐and‐error methods, theoretical simulations are strategically used as an efficient strategy to predict the photonic properties of W_1.33_C *i*‐MXene. The results of theoretical simulations suggest that W_1.33_C *i*‐MXene possesses strong absorbance in the NIR region. Experimentally, we systematically assess the biocompatibility, degradability and light‐to‐heat conversion performance of the W_1.33_C *i*‐MXenes as a photothermal agent (PTA) by laser activation in NIR range at both the first (NIR I) and second (NIR II) biowindow. Bovine serum albumin (BSA)‐modified W_1.33_C *i*‐MXene (W_1.33_C‐BSA) exhibits an excellent photothermal‐conversion efficacy both in vitro and in vivo. Considering the acidic and hypoxic tumor microenvironment (TME), the as‐prepared W_1.33_C‐BSA nanosheets with pH‐dependent biodegradation properties are comparatively stable in acidic environments and can rapidly degrade under physiological conditions. Therefore, W_1.33_C‐BSA nanosheets could efficiently accumulate into tumors for inducing longer tumor retention and would be rapidly degraded and excreted without obvious systemic toxicity. Taking advantage of superior multimodal‐imaging properties for computed tomography (CT)/PA/photothermal imaging, we further demonstrate that W_1.33_C‐BSA nanosheets can be used as a robust, versatile, and noninvasive imaging contrast agent (CA) to monitor cancer therapy in vivo. In addition, we further reveal the underlying biomedical mechanism of PTT as enabled by W_1.33_C *i*‐MXene. Joint analysis of proteome and transcriptome uncovers that W_1.33_C‐BSA nanosheets‐induced PTT activate the “complement and coagulation cascades” signaling pathway, resulting in cell membrane rupture and cell lysis. Therefore, this work presents a distinct kind of *i*‐MXenes with ordered vacancies that possess fast biodegradation and rapid excretion, accompanying with efficient tumor retention as well as theranostic functionalities, which significantly broadens the biomedical applications of 2D MXenes.

## 2 Results and Discussion

### 2.1 Synthesis and Characterization of 2D W_1.33_C‐BSA Nanosheets

**Figure 1:**
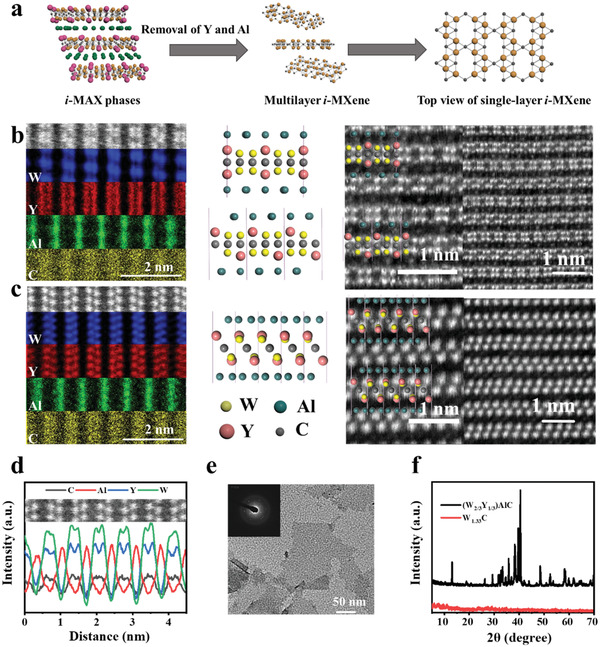
a) Scheme of exfoliation process for 2D W_1.33_C nanosheets. b) High‐resolution STEM (HSTEM) images and corresponding schematic in [110] direction. c) HSTEM images and corresponding schematic in [100] direction. d) EDX linear‐scanning along [100] direction (the line depicted in inset), mapping the W, Y, Al, and C element signals of (W_2/3_Y_1/3_)_2_AlC. e) TEM image and corresponding SAED (inset image) of W_1.33_C. f) XRD patterns of (W_2/3_Y_1/3_)_2_AlC *i*‐MAX and W_1.33_C *i*‐MXene.

The quaternary *i*‐MAX phase (W_2/3_Y_1/3_)_2_AlC bulk was fabricated by sintering W, Y, Al, and C powders under argon (Ar) atmosphere up to 1450 °C and then immersed into the etchant solution mixing fluoride (LiF, 4 g) with hydrochloric acid solution (HCl, 60 mL, 12 m) to remove both Y and Al layers (**Figure** **1a**). The as‐obtained (W_2/3_Y_1/3_)_2_AlC powders were initially characterized by scanning electron microscopy (SEM). The corresponding SEM images of (W_2/3_Y_1/3_)_2_AlC and element mapping reveal the presence and distribution of W, Y, Al, and C (Figure S1a, Supporting Information). After etching and exfoliation treatment, the compact layers expanded, leading to the accordion‐like multilayered topology, further demonstrating the exfoliation of (W_2/3_Y_1/3_)_2_AlC and selective removal of Y/Al for the production of 2D W_1.33_C *i*‐MXene (Figure S1b, Supporting Information). To explore more detailed microarchitecture information, the high‐angle annular dark field (HAADF) imaging in scanning transmission electron microscopy (STEM) was acquired in the [110] and [100] direction of *i*‐MAX phase (Figure 1b,c). The W/Y/C atoms are on the same layer and alternately arranged with Al atoms. In addition, according to the HAADF imaging and corresponding energy dispersive X‐ray spectrum (EDX) linear‐scanning analysis (Figure 1d), the mean distance between two adjacent Al atom is 0.68 nm. Transmission electron microscopy (TEM) images exhibit the ultrathin structure of exfoliated W_1.33_C nanosheets with a typical lateral size of ≈150 nm (Figure 1e). From EDX elemental mapping results of W_1.33_C *i*‐MXene (Figure S2, Supporting Information), it is concluded that both the Y and Al atoms were selectively etched.

The X‐ray diffraction (XRD) pattern reveals that the (W_2/3_Y_1/3_)_2_AlC *i*‐MAX phase was successfully synthesized (Figure 1f, black curve). After etching, the peak intensities from (W_2/3_Y_1/3_)_2_AlC bulk decrease significantly, indirectly demonstrating that the bulk crystal has been converted to W_1.33_C *i*‐MXenes. The thickness of the fabricated W_1.33_C nanosheets is determined using atomic force microscopy (AFM) to be around 0.25 nm (Figure S3, Supporting Information). To improve the stability in the physiological environment for further biomedical application, BSA as a bioactive macromolecule was used to modify the surface of W_1.33_C nanosheets through hydrogen bonding and/or van der Waals attractive interactions. The successful synthesis of W_1.33_C‐BSA nanosheets is confirmed by Fourier transform infrared (FTIR) spectroscopy (Figure S4, Supporting Information), revealing that two distinct and strong absorption peaks at ≈1542 and 1665 cm^−1^ , assigning to the stretching vibrations of —NO_2_ and —C═O— in BSA.^[^ ^18^ ^]^ As expected, the obtained W_1.33_C‐BSA nanosheets exhibit high dispersity in different physiological solvents such as phosphate buffer saline (PBS) and cell culture media. Furthermore, the average hydrodynamic diameters of W_1.33_C‐BSA nanosheets is around 190 nm, which is a little bigger than bare W_1.33_C nanosheets (≈160 nm), indicating the successful BSA modification (Figure S5, Supporting Information). Meanwhile, the change of zeta potential is induced by the enhanced superficial hydrophilicity and bio‐macromolecular charge‐shielding effect attached to the surface (Figure S6, Supporting Information). Owing to the successful surface modification of the W_1.33_C nanosheets with BSA, the W_1.33_C‐BSA nanosheets exhibit favorable stability in various physiological conditions such as saline, PBS, Dulbecco's modified Eagle medium (DMEM), and whole blood diluent (Figure S7, Supporting Information).

### 2.2 Optical Properties of W_1.33_C Nanosheets (*i*‐MXenes)

**Figure 2:**
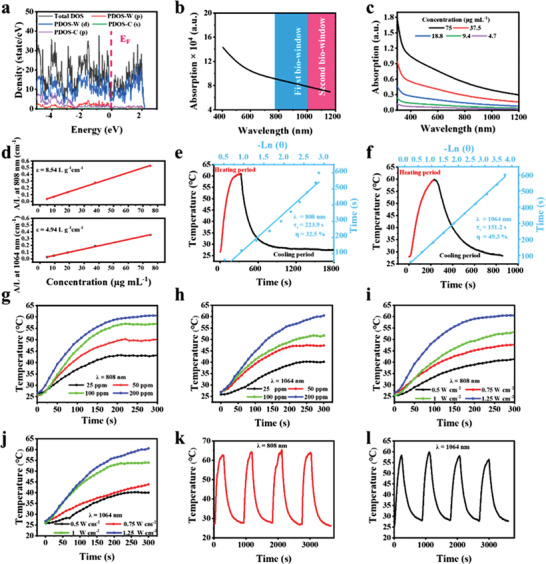
a) The total and local DOS of W_1.33_C *i*‐MXene. b) Calculated photo‐absorption spectrum of W_1.33_C *i*‐MXene. c) Absorption spectra of W_1.33_C nanosheets dispersion at various concentrations (4.7, 9.4, 18.8, 37.5, and 75 µg mL^−1^). d) Mass extinction coefficient of W_1.33_C nanosheets at *λ* = 808/1064 nm. e) Photothermal conversion efficiency (PCE) at 808 nm irradiation. f) PCE at 1064 nm laser irradiation. Concentration‐dependent temperature‐changing curves under g) 808 nm and h) 1064 nm laser illumination. Photothermal heating curves of W_1.33_C nanosheets solutions irradiated by a series of power densities of i) 808 nm and j) 1064 nm laser. Heating curve of W_1.33_C nanosheets dispersion for four laser on/off cycles irradiated by k) 808 nm and l) 1064 nm laser at 1.25 W cm^−2^.

The strong light absorption of PTA is essential to guarantee efficient light‐to‐heat conversion. The absorption spectra could be simulated by the typical density functional theory (DFT) calculations. Based on the first‐principle calculation, the density of states (DOS) reveals that W_1.33_C *i*‐MXene exhibits metallic properties (**Figure** **2a**). Notably, the simulated optical absorption spectrum acquired on W_1.33_C *i*‐MXene exhibits a broadband absorption in NIR region, which offers high potential for photonic nanomedicine. The further experimental results show that W_1.33_C *i*‐MXene nanosheets have broadband absorption across both NIR I and II biowindows, consistent with the theoretically calculated spectrum, implying their potential as a PTA candidate (Figure 2b,c). The results reveal that the extinction coefficients (*ε*) of W_1.33_C nanosheets are 8.54 and 4.94 L g^−1^ cm^−1^ at 808 and 1064 nm, respectively(Figure 2d). Furthermore, the photothermal‐conversion efficiency (*η*), another essential parameter for the photothermal performance of W_1.33_C nanosheets is 32.5% (NIR I, 808 nm) and 49.3% (NIR II, 1064 nm), indicating that W_1.33_C nanosheets could serve as a powerful PTA for their applications in PTT in the bio‐windows, especially in NIR II biological window (Figure 2e,f). Especially, the W_1.33_C nanosheet dispersion exhibits quick temperature increase in a concentration‐dependent manner from 26.0 to 60.5 °C under NIR I laser irradiation and from 26.3 to 60.9 °C under NIR II laser irradiation (Figure 2g,h), respectively. In addition, the photothermal temperature of W_1.33_C nanosheet dispersion can also be elevated with the increase of laser radiation power density (Figure 2i,j), further demonstrating the controllable photothermal performance of 2D W_1.33_C nanosheets. For the photothermal stability, it is demonstrated that no significant change in the thermal performance was recorded during four laser on/off cycles (Figure 2k,l).

Afterward, to confirm the superiority of W_1.33_C‐BSA nanosheets in NIR II photothermal performance, the deep‐tissue photothermal‐penetration experiments were further carried out using 4T1 tumor‐bearing mice where 4 mm‐thickness chicken breast tissue is on the top of the tumor area. As expected, the highest increase in temperature (△*T*) after illumination under NIR II laser for 10 min is 13.7 °C, whereas the △*T* is only 6.5 °C under NIR I laser irradiation for 10 min (Figure S8, Supporting Information). Therefore, these results verify that W_1.33_C‐BSA nanosheets could serve as a high‐performance photothermal converter for PTT treatment, featuring high photothermal‐conversion efficiency and favorable tissue penetration, especially in NIR II biowindow.

### 2.3 Degradation Behavior of W_1.33_C *i*‐ MXene

Rapid biodegradation of PTA could reduce long‐term toxicity and guarantee high in vivo biosafety. To evaluate the degradation behavior of W_1.33_C‐BSA nanosheets, a series of experiments were executed by observing the ultraviolet–visible–NIR (UV–vis–NIR) absorption spectrum changes of W_1.33_C‐BSA nanosheets in PBS and fetal bovine serum at different pHs (Figures S9 and S10, Supporting Information). It could be visually found that W_1.33_C‐BSA nanosheets exhibit a significant pH‐dependent degradation effect. W_1.33_C‐BSA nanosheets rapidly degrade in basic circumstances, while they are comparatively stable under the acidic environment, implying that W_1.33_C‐BSA nanosheets could deliver more durable therapeutic effects in the tumor microenvironment with weak acidic and hypoxia conditions. Notably, the estimated pH within the 4T1 breast cancer tumor is about 6.0–6.5.^[^ ^19^ ^]^ We thus infer that W_1.33_C‐BSA nanosheets degrade more slowly in tumour compared to normal tissue.

For further investigation of W_1.33_C‐BSA nanosheets in blood degradation, the PBS containing 10% mouse blood UV–vis–NIR absorption spectra over varied incubation durations (2, 4, 8, 12, and 24 h) were performed. The degradation of W_1.33_C‐BSA nanosheets in blood was revealed to be a time‐dependent procedure that exhibits an increase with the extension of time (Figure S11, Supporting Information). Additionally, TEM observation was used to monitor the structure changes during the degradation process (Figure S12, Supporting Information). It is found that the significant surface oxidation on the layer structure of partial nanosheets takes place at the initial stage, and then the severe surface oxidation induces a slight breakup of planar dimensions on the layer structure of W_1.33_C‐BSA nanosheets. Finally, the complete decomposition for planar dimensions of W_1.33_C‐BSA nanosheets occurred at the final stage. The intracellular degradation behavior and structural evolution of W_1.33_C‐BSA nanosheets were further evaluated on the cellular level (Figure S13, Supporting Information). Initially, W_1.33_C‐BSA nanosheets could be effectively endocytosed into cancer cells and surface oxidation occurred within 1 d. Subsequently, prolonged incubation lead to severe surface oxidation and almost complete breakdown of W_1.33_C‐BSA nanosheets in planar dimensions after 3 d, indicating that the nanosheets underwent significant biodegradation.

PA imaging combines the advantages of optical and ultrasound imaging. The intensity of PA signal is proportional to the absorbed optical energy and the thermoelastic energy of PTA. When W_1.33_C‐BSA nanosheets degraded, the light absorption and the thermoelastic performances of W_1.33_C‐BSA would also decrease, which leads to a decrease in the PA signal. Therefore, PA imaging has been applied to monitor the in vivo degradation of W_1.33_C‐BSA nanosheets in muscle and tumors. Intratumorally or intramuscularly injections of W_1.33_C‐BSA nanosheets were administered to 4T1 tumor‐bearing mice and PA imaging was acquired at varied time points of post‐injection (Figure S14, Supporting Information). As expected, compared to original PA signals in muscles, the intensity of PA signal is only about 30% of their original signals 1 h later. On the contrary, 60% of original PA signals were observed in the tumor within 1 h, which demonstrates the obviously slower degradation rate of W_1.33_C‐BSA nanosheets in tumor than that in muscle. Therefore, these biodegradable W_1.33_C‐BSA nanosheets would enable their longer tumor retention and harmless degradation during exerting their therapeutic functions. The clearance of W_1.33_C‐BSA nanosheets was monitored by measuring W contents in urine and feces of mice using inductively coupled plasma atomic‐emission spectroscopy (ICP‐AES) (Figure S15, Supporting Information). It is found that the W contents were high in urine and feces during the 48 h of post‐injection, demonstrating that W_1.33_C‐BSA nanosheets could be quickly cleared from the mouse via renal and fecal routes. The biodistribution of W_1.33_C‐BSA nanosheets in different tissues was investigated at different time points. The results show that W_1.33_C‐BSA nanosheets mainly accumulate in the liver. The intratumor passive accumulation efficiency based on W has been quantified as 4.87%, 3.42%, and 3.20% ID g^−1^ in 4, 24, and 48 h, respectively, which is competitive compared to current 2D nanomaterials used in cancer(Figure S16, Supporting Information). Taken together, it could be deduced that W_1.33_C‐BSA nanosheets would be a biodegradable PTA, which could not only be relatively stable in tumors but also enable rapid excretion for avoiding long‐term in vivo toxicity.

### 2.4 In Vitro Photothermal Cancer Cell Ablation and Endocytosis

**Figure 3:**
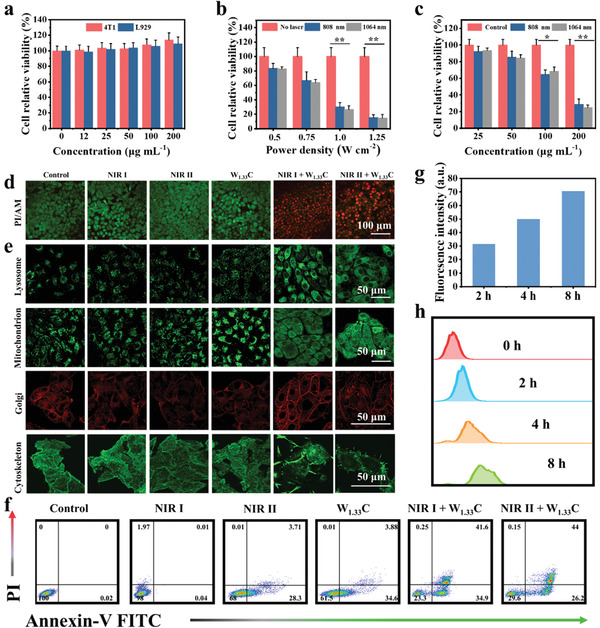
a) Relative survival rate of 4T1 and L929 cells after incubated with W_1.33_C‐BSA nanosheets dispersion at elevated concentrations for 24 h . Relative survival rate of 4T1 cells treated with W_1.33_C‐BSA nanosheets dispersion at b) different power densities and c) various concentrations under 808/1064 nm laser irradiation for 10 min (*n* = 5, mean ± SD). d) CLSM images of 4T1 cells after various treatments, followed by staining with calcein‐AM (green) and PI (red). e) CLSM images of cellular organelles, including LysoTracker DND‐26 stained Lyso (green), MitoTracker FM stained Mito (green), Gi‐Tracker stained Gi (red), and Alexa Fluor 488 conjugated phalloidin stained Cyto (green). f) FCM apoptosis assay of 4T1cells after different treatments with the staining of annexin V‐FITC and PI. g) Corresponding FITC fluorescence signals of CLSM images of 4T1 cells incubated with FITC‐labeled W_1.33_C‐BSA nanosheets for 2, 4, and 8 h. h) Cellular uptake amounts of W_1.33_C‐BSA nanosheets as analyzed by FCM, and the corresponding FITC intensity of 4T1 cells incubated with FITC‐labeled W_1.33_C‐BSA nanosheets for 0, 2, 4, and 8 h.

For in vitro cytotoxicity assessment, W_1.33_C‐BSA nanosheets dispersion was incubated with both cancer cell (4T1 mouse breast cancer cell line) and normal cell lines (L929 mouse fibroblast cell line) at pre‐supposed concentrations. As shown **Figure** **3a**, negligible cytotoxicity was detected from both cell lines even at the concentration up to 200 µg mL^−1^ after 24 h co‐incubation, which signifies that these W_1.33_C‐BSA nanosheets possess low cytotoxicity. Furthermore, the in vitro photothermal ablation capability of W_1.33_C‐BSA nanosheets was also assessed via Cell Counting Kit‐8 (CCK‐8) assay. It is revealed that as power density and concentration W_1.33_C‐BSA nanosheets increased, more cancer cells are killed upon NIR laser illumination (Figure 3b,c). The survival rate is down to 29% or 28% when 4T1 cells are irradiated with NIR I or NIR II for 10 min, respectively

To better visualize the treatment performance of W_1.33_C‐BSA nanosheets on 4T1 cells in vitro, co‐stained imaging of calcein‐AM (green) and propidine iodide (PI, red) was acquired using confocal laser scanning microscopy (CLSM) (Figure 3d). It is found that the majority of 4T1 cells are killed after treatment with W_1.33_C‐BSA nanosheets under NIR I or II laser illumination, while the 4T1 cells in other groups ( control, W_1.33_C‐BSA nanosheets only, NIR I only, and NIR II only) are not significantly influenced. Generally, the cell death is concomitant with the alternation of the cellular morphology, structure and organelle function. Cellular organelles, which mainly include lysosomes, mitochondria, Golgi apparatus, endoplasmic reticulum, and cytoskeleton, play vital roles in regulating cellular activities and survival.^[^ ^20^ ^]^ Therefore, after W_1.33_C‐BSA nanosheets‐mediated PTT treatment, detailed subcellular structure evolution was further tracked by corresponding cellular structure fluorescent probe staining (Figure 3e and Figure S17, Supporting Information). For lysosome, punctate green signals in the cytoplasm are observed from the imaging of 4T1 cells in control groups. Adversely, disseminated green fluorescence staining appears throughout the cytoplasm in treatment groups implying that W_1.33_C‐BSA nanosheets are able to initiate the damage of lysosomes under both NIR I and NIR II laser irradiation. Similarly, hierarchical mitochondrial fragmentation, Golgi apparatus destruction, cytoskeleton damages, and endoplasmic reticulum shrunken induced by hyperthermia are discovered. By contrast, there is negligible morphology changes in mitochondria, Golgi apparatus, cytoskeleton, and ER of 4T1 cells incubated with W_1.33_C‐BSA nanosheets or under laser irradiation alone.

Subsequently, the quantification ratio of viable and apoptotic cells during the process of PTT was revealed by flow cytometry (FCM) apoptosis assay (Figure 3f). It is found that the apoptotic cell populations are 76.5% (34.9% for the early apoptotic ratio, 41.6% for late apoptotic ratio) and 70.2% (consisting of the early and late apoptotic ratio of 26.2% and 44%, respectively) for the group of W_1.33_C‐BSA + NIR I and W_1.33_C‐BSA + NIR II, respectively. Comparatively, 4T1 cells incubated with W_1.33_C‐BSA nanosheets groups or only laser irradiation remain at very high survival rates, indicating the high biocompatibility of W_1.33_C‐BSA nanosheets. Cell cycle analysis was further performed using FCM to obtain more insights into the anti‐cancer effect of PTT. After co‐incubated with W_1.33_C‐BSA nanosheets plus NIR I or II laser illumination, an obvious increment in the percentage of 4T1 cells at the sub‐G1 phase (Figure S18, Supporting Information) is observed. Conversely, the percentage of 4T1 cells in the sub‐G1 phase of the other groups shows no significant increase. These results indicate that W_1.33_C‐BSA nanosheets‐triggered hyperthermia resultes in significant cell‐cycle inhibition and 4T1 cells apoptosis. Efficient cellular endocytosis of the nano‐agents is a prerequisite for anticancer therapy. To understand the mechanism of in vitro cancer cell inhibition by W_1.33_C‐BSA nanosheets, the intracellular uptake of W_1.33_C‐BSA nanosheets was further assessed. Because W_1.33_C‐BSA nanosheets show no autofluorescence and fluorescein isothiocyanate (FITC)‐labeled W_1.33_C‐BSA nanosheets exhibite a strong fluorescence signal at 525 nm (Figure S19, Supporting Information), the FITC‐labeled W_1.33_C‐BSA nanosheets were used to observe the intracellular uptake of W_1.33_C‐BSA nanosheets. CLSM images clearly show the efficient intracellular uptake of W_1.33_C‐BSA nanosheets (FITC conjugated BSA, green) after the co‐incubation for 8 h (Figure 3g and Figure S20, Supporting Information). As shown in Figure 3h, the internalization amount of W_1.33_C‐BSA nanosheets was determined by FCM over 8 h. The mean fluorescence intensities exhibit time‐dependent increment. Especially, the green fluorescence of W_1.33_C‐BSA‐FITC rarely overlapped with the red fluorescence of LysoTracker red and MitoTracker red when 4T1 cells were co‐incubated with W_1.33_C‐BSA nanosheets, indicating that these materials scattered within the cytoplasm (Figures S21 and S22, Supporting Information).

### 2.5 In Vivo Anticancer Activity

A systematic investigation of in vivo toxicology for W_1.33_C‐BSA nanosheets was further conducted before subsequent treatment. Healthy Kunming mice were raised and classified into four groups based on the different dosages of W_1.33_C‐BSA nanosheets injected intravenously. The mice after different treatments were euthanized on the 28th day. It has been found that there is no abnormality in the blood routine and biochemical indexes in four groups (Figure S23, Supporting Information). In addition, no significant histological abnormality is recorded in major organs after 28 d feeding via the hematoxylin and eosin (H&E) staining (Figure S24, Supporting Information).

**Figure 4:**
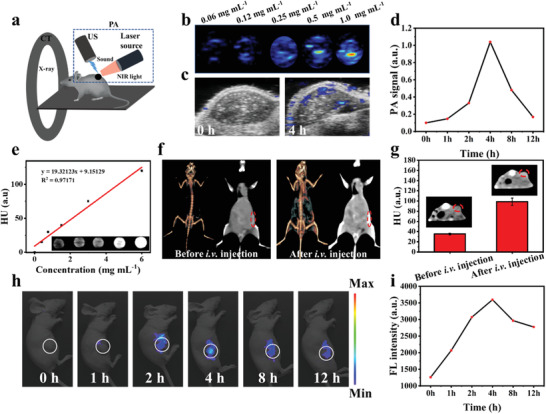
a) Schematic illustration of W_1.33_C‐BSA nanosheets enabling in vivo multimodal imaging and b) in vitro PA images of the W_1.33_C‐BSA at elevated concentrations (0.06–1 mg mL^−1^). c) In vivo PA images and d) quantitative changes in PA signals of tumor region. e) In vitro CT contrasts and CT images (inset) of W_1.33_C‐BSA nanosheet solutions at varied concentrations and corresponding CT intensities. f) In vivo CT contrasts before and after i.v. injection. g) Coronal plane images (inset) and corresponding signal intensities of CT imaging before and after intravenous injection. h) In vivo fluorescence images and i) corresponding quantified fluorescence intensity changes of tumor tissues at different time points (0, 1, 2, 4, 8, and 12 h) after i.v. injection with FITC‐labeled W_1.33_C‐BSA.

In respect of the W_1.33_C‐BSA nanosheets have strong NIR‐absorbance in the NIR region, W_1.33_C‐BSA nanosheets are expected to be an ideal PA probe(**Figure** **4a**). To confirm this, PA imaging was further performed using W_1.33_C‐BSA nanosheets as a probe. As shown in Figure 4b, the signal intensity of the PA images increases linearly with the increasing concentration of W_1.33_C‐BSA nanosheets. Subsequently, 4T1‐tumor‐bearing mice were intravenously injected with W_1.33_C‐BSA nanosheets and PA images were recorded at pre‐assigned time points. The PA intensity value of W_1.33_C‐BSA nanosheets after 4 h accumulation in tumor site increases from 0.1 to 1.04 a.u. (Figure 4c,d), which is ten fold stronger than that before administration. After 4 h, the PA intensity starts to decrease, suggesting the degradation or metabolism of W_1.33_C‐BSA nanosheets in the tumor section.

Another intriguing property of W_1.33_C‐BSA nanosheets results from high atomic number element W (*Z* = 74), which enables them as a contrast agent (CA) for CT imaging.^[^ ^21^ ^]^ Therefore, contrast‐enhanced CT imaging was further explored using W_1.33_C‐BSA nanosheets as CAs. As shown in Figure 4e, the Hounsfield unit (HU) values of CT image enhance linearly with elevation concentration of W_1.33_C‐BSA nanosheets. 4T1 subcutaneous tumor‐bearing mice were administrated intravenously with W_1.33_C‐BSA nanosheets at concentration 20 mg kg^−1^ for in vivo CT imaging. After 4 h post‐injection of W_1.33_C‐BSA nanosheets, the CT signal at the tumor site significantly increase from 37.5 ± 2.6 to 98.2 ± 14.6 HU (Figure 4f,g). Therefore, this result provides a strong evidence that W_1.33_C‐BSA nanosheets could be used as an efficient CA for contrast‐enhanced CT imaging.

**Figure 5:**
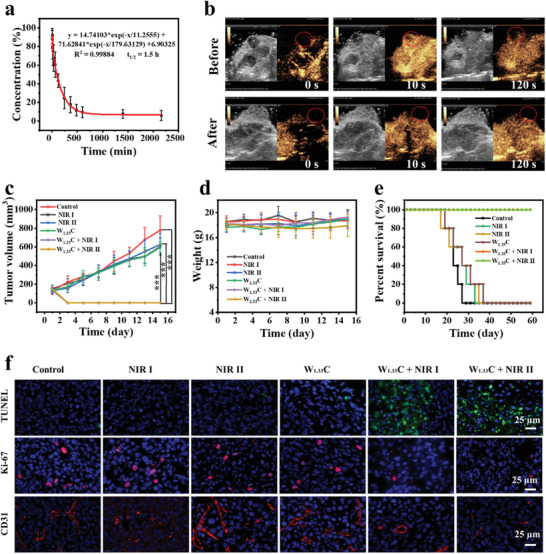
a) Blood circulation curve of W_1.33_C‐BSA after intravenous injection and *T*_1/2_ was calculated to be about 1.5 h. b) CEUS images before and after PTT. c) Tumor‐growth curves after different treatments (*n* = 5, mean ± SD) (control, NIR I only, NIR II only, W_1.33_C‐BSA only, W_1.33_C‐BSA + NIR I, and W_1.33_C‐BSA + NIR II). (d) The curve of mice weight changes during different treatments (*n* = 5, mean ± SD). e) Analysis of the survival of mice during different treatments. f) The corresponding TUNEL, Ki‐67, and CD31 staining for various changes in tumor section (scale bar = 25 µm).

The blood circulation and biodistribution of W_1.33_C‐BSA nanosheets were assessed before the in vivo photonic hyperthermia experiments. The biodistribution of W_1.33_C‐BSA nanosheets was evaluated through in vivo fluorescence imaging system at the prespecified time, in which the fluorescence signal was generated by the conjugated FITC. As shown in Figure 4h,i, the in vivo fluorescence signal changes can be detected obviously with the prolonged time, showing that the strongest signal in the tumor sites is at 4 h after intravenous (i.v.) injection and gradually decreases thereafter. It suggests that W_1.33_C‐BSA nanosheets can be accumulated within tumor and excreted from the body to avoid long‐term toxicity. The morphology of nanomaterials could not only be a factor influencing the biodistribution in living bio‐system but also impact the behavior of cellular uptake, which might be ascribed to the surface area of nanomaterials in contact with cell films and the distortion energy of the cellular films during uptake. Although the contribution of shape to biology in every type of nanostructure might be different, the regulation of this property can be investigated to enhance the performance of nanomaterials‐mediated PTT. Additionally, the circulation of W_1.33_C‐BSA nanosheets in blood was initially analyzed and the half‐time of W_1.33_C‐BSA nanosheets is calculated to be about 1.5 h (**Figure** **5a**).

The in vivo therapeutic performance was further evaluated on 4T1subcutaneous tumor‐bearing mice, which were randomized into six groups, including control (G I), NIR I only (G II), NIR II only (G III), W_1.33_C‐BSA only (G IV), W_1.33_C‐BSA + NIR I (G V), and W_1.33_C‐BSA + NIR II (G VI). 4T1 tumor‐bearing mice were anesthetized and performed with different treatments when the volume of the tumor reached ≈150 mm^3^. Meanwhile, the temperature of tumor site was measured by infrared thermal camera. It is worth noting that the tumor site temperatures of G V increased by 23 °C. Meanwhile, in G VI, the tumor‐site temperature increased by 25 °C. Comparatively, the tumor temperature of the mice in G II and G III only increased by 5 °C within 5 min (Figure S25, Supporting Information). In particular, the contrast‐enhanced ultrasound (CEUS) was adopted to precisely evaluate the degree and margination of ablation by real‐time monitoring the microcirculation perfusion of the photothermal ablation foci. As shown in Figure 5b, there is a clear edge between ablated tissues and normal tissues, indicating that W_1.33_C‐BSA nanosheets mediated PTT allows for precise image‐guided treatment. Remarkably, the tumors in two treated groups (G V and VI ) disappeared, leaving black scars in 2 d after PTT treatment. Comparatively, the tumor size of mice in the groups of G I–IV grew up rapidly, indicating no tumor inhibition during these treatments (Figure 5c). Additionally, no significant bodyweight fluctuation is observed during the period of treatment, indicating that W_1.33_C‐BSA nanosheets could be applied as an effective PTA without adverse effects on mice health (Figure 5d). It is gratifying that tumors in G V and VI were entirely eliminated without further reoccurrence and the 60‐d‐survival rate of these groups was 100%, much higher than that in G I–IV (Figure 5e). The results of terminal deoxynucleotidyl transferase dUTP nick end labeling (TUNEL) assay show that apoptotic cells in the G V and VI are significantly more than that in other groups. As further demonstrated by Ki‐67 antibody staining, G V and VI showed a strong inhibitory effect on cell proliferation, unlike the other four groups which exhibited almost no effects on the proliferation of cancer cells. Additionally, we found a significant reduction of CD31^+^ vasculatures in two treated groups (G V and VI), while the other four groups showed almost negligible changes on cancer cells (Figure 5f).

**Figure 6:**
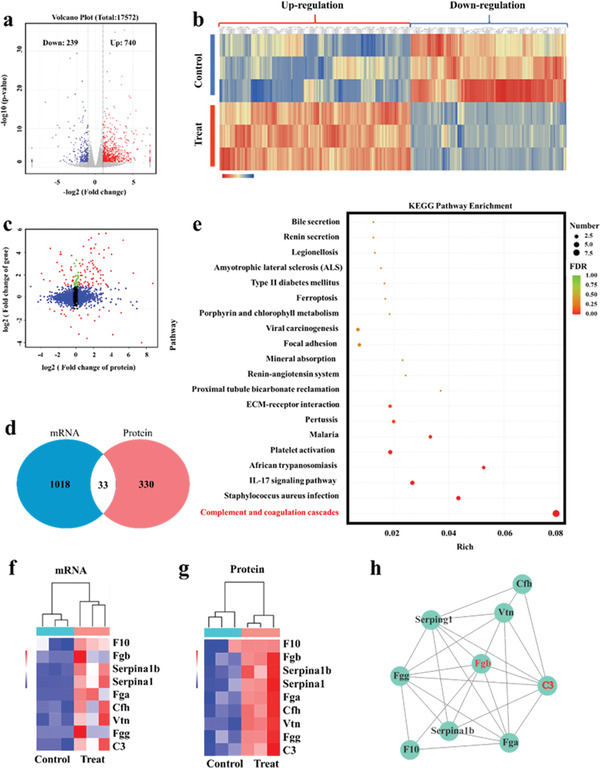
Joint analysis of transcriptome and proteome analysis. a) Volcano plot of genes altering after the treatment of PTT (*P* < 0.05, |fold change| ≥ 2). b) Heat map of differential proteins (*P* < 0.05), including 200 downregulated proteins and 163 upregulated proteins following PTT treatment. c) Analysis of the correlation between protein and gene expression (red for both in transcriptome and proteome; blue for only in proteome; green for only in transcriptome; black for either transcriptome or proteome). d) Venn diagram of the “mRNA” and “protein.” e) KEGG enrichment analysis for studying the underlying pathways PTT treatment. Heat map for showing the differentially expressed genes of differential expression of f) related transcripts and g) proteins in the “complement and coagulation cascades” pathway. h) PPI network of these proteins in the “complement and coagulation cascades” pathway.

To further explore the underlying bio‐mechanism of W_1.33_C‐BSA nanosheets‐induced PTT, transcriptomic and proteomic analysis were performed to study the gene and protein changes of tumor on nude mice with different treatments. Transcriptome sequencing results indicate that there are 979 differential genes (fold change ≥ 1 and *p* < 0.05) between control and PTT treatment groups, including 740 upregulated genes and 239 downregulated genes (**Figure** **6a**). Protein, the executor of life functions, plays an important role in regulating biological process. Thus, we performed label‐free quantitative proteomics technology (LFQPT) to explore the protein changes in tumor site on nude mice with different treatments. The results of heatmap analysis (Figure 6b) of the LFQPT‐based proteomics show 4830 differential proteins, where the 200 downregulated proteins (|fold change| ≥ 2) and 163 upregulated proteins (|fold change| ≥ 2) represent a wide range of biological changes due to PTT treatment.

It is well known that the process from mRNA to protein involves a complete set of precise expression control mechanisms, such as transcription regulation, post‐transcriptional regulation, translation regulation, post‐translational regulation, etc. Therefore, simultaneously detecting the expression of mRNA and protein and conducting joint analysis are highly important to comprehensively explore the biological disease mechanism. Through joint analysis of proteome and transcriptome, we could implement the complementation and integration of mRNA and protein, and conduct a comprehensive analysis of the mechanism in a specific state of the organism. To comprehensively explore the underlying biological mechanism of the effects induced by 2D W_1.33_C‐BSA nanosheets, we performed a joint analysis of proteome and transcriptome. The relationship between gene and protein expression is exhibited by the volcano plot (Figure 6c). As shown in Figure 6d, there are 33 categories of different proteins and transcripts with corresponding relationships, including 30 co‐upregulated and 3 co‐downregulated genes and proteins. Next, we performed functional and pathway enrichment analysis on the final differential genes corresponding to above‐mentioned differential proteins and transcriptome. It is clear that more of identified proteins are concentrated in cellular components (especially extracellular components) in Gene Ontology (GO) enrichment analysis, suggesting that the essential changes taken place in the extracellular region (Figure S26, Supporting Information). Various components of the extracellular matrix (ECM) are intimately involved in cell recognition, adhesion, proliferation, and migration, especially in tumor‐tissue stress states.^[^ ^22^ ^]^ To explore potential signaling pathways after PTT treatment, the identified proteins were annotated using the Kyoto Encyclopedia of Genes and Genomes (KEGG). As shown in Figure 6e, the “complement and coagulation cascades” signaling pathway was the most salient pathway among the top 20 terms of enriched KEGG pathway.

Complement and coagulation are essential for an adequate congenital response to injury. The system could be activated by exposure to alleged danger signals or damage related molecular patterns (e.g., pathogens and damaged host cells). With exposure to pathogens or damaged cells, C3 is cleaved to produce a series of complements, including C3b, C3a, C5a, C5b, etc., of which C5b‐9 is the primary component needed for the spontaneous formation of the membrane attack complex (MAC), which aggregates and invokes cleavage of the target cell. Thus, C3 plays an important role in this pathway, which is consistent with the enrichment results in this study. In this work, PTT induced hyperthermia induced can lead to tumor vascular damage, which could be seen as a danger signal that activates the complement and coagulation cascade. On the one hand, hyperthermia can lead to the tumor vascular damage, activating the exogenous thrombin pathway, converting fibrinogen (Fgb) into fibrin and then blocking tumor blood vessels, which ultimately starve tumor to death. In addition, after PTT treatment, a set of membrane attack complexes on the surface of cancer cells, formed by a cascade reaction in the classical pathway, lead to the entry of water into the cells due to the great difference in osmotic pressure, which eventually leads to the rupture of the cell membrane and cell lysis (Figure 6f–h).

## 3 Conclusions

In summary, the distinct 2D W_1.33_C nanosheets (*i*‐MXene) with ordered divacancies and desirable biocompatibility/biodegradability were successfully constructed for cancer nanotheranostics. These intriguing MXene nanosheets have been demonstrated to feature distinct PTA functions for multimodal imaging (PA/CT/photothermal imaging) and photonic tumor hyperthermia. Especially, theoretical simulation has been employed to predict W_1.33_C nanosheets with a strong NIR absorption band. After surface modification with BSA, these ultrathin 2D W_1.33_C‐BSA nanosheets exhibit high biocompatibility, rapid degradation, and high photothermal‐conversion efficiency. Importantly, these W_1.33_C‐BSA nanosheets could be rapidly cleared from normal organs, but their unique pH‐responsive characteristics allowed them to enrich and remain longer at tumor sites. Benefiting from the superior X‐ray attenuation ability and high NIR absorbance of W_1.33_C‐BSA nanosheets, remarkably enhanced in vivo CT and PA multimodal imaging of tumors has been executed. Especially, highly effective in vivo PTT by W_1.33_C‐BSA nanosheets of tumor has been systematically assessed on tumor‐bearing mice. The underlying mechanism and regulation factors for the engineered W_1.33_C‐BSA nanosheets induced PTT are also revealed via genomics and proteomics. Therefore, this work significantly expands the biomedical applications of *i*‐MXene‐based nanomedicines

## 4 Experimental Section

### Synthesis of (W_2/3_Y_1/3_)_2_AlC Powders (*i*‐MAX)

The powders of W, Y Al and C were mixed at a molar ratio of 1.33:1.33:1:1 to prepare (W_2/3_Y_1/3_)_2_AlC MAX‐phase ceramics. After that, the hybrid powders were cool‐pressed into the disc, and then heating to 1450 °C with a rate of 8 °C min^−1^ for 2 h with flowing argon (Ar) atmosphere. After cooling to room temperature, the sample was gently pulverized to yield the final product ((W_2/3_Y_1/3_)_2_AlC *i*‐MAX phase powders).

### Synthesis of W_1.33_C Nanosheets (*i*‐MXenes)

The as‐obtained (W_2/3_Y_1/3_)_2_AlC precursor (2 g) was immersed into HCl aqueous solution (60 mL, 12 m) with LiF (4 g), and stirred for 48 h at 35 °C in an oil bath. After etching, the products were collected by centrifugation, adding HCl (50 mL) and shaking manually for 1 min, followed by centrifugation for 1 min (5000 rpm), and this process was repeated three times. The etched W_1.33_C nanosheets were then washed with deionized water (ddH_2_O) for several times until delamination occurred to obtain ultrathin W_1.33_C nanosheets. In order to inhibit the possible oxidation, the HCl and ddH_2_O were degassed for 30 min.

### Surface Modification of W_1.33_C Nanosheets (W_1.33_C‐BSA)

The as‐prepared nanosheets were water‐soluble, while it was not stable in the biological environment without further modification. To improve stability in physiological solution, W_1.33_C nanosheets were blended with BSA aqueous solution at room temperature with stirring and maintained in vacuum for 2 h. Then, the as‐prepared W_1.33_C‐BSA nanosheets were gathered by centrifugation and washed several times with degassed ddH_2_O to remove excess and free BSA. Finally, the acquired W_1.33_C ‐BSA sediment was redispersed in degassed DI water and stored at 4 °C.

### In Vivo Deep‐Tissue Photothermal‐Penetration Exam in Both NIR I and NIR II Biowindows

To simulate the clinical situation, the in vivo deep‐tissue photothermal capability of the W_1.33_C‐BSA nanosheets was evaluated 4T1 breast tumor‐bearing mice model with the tumor areas covered by a 4 mm thick chicken breast tissue. The experimental conditions were as follows: laser wavelength, 808/1064 nm; laser power density, 1.5 W cm^−2^; duration time, 5 min. Meanwhile, IR thermal camera was used to record the temperature change.

### In Vitro Cell Toxicity of W_1.33_C‐BSA Nanosheets

4T1 cells and L929 cells were used to assess the cytotoxicity of W_1.33_C‐BSA nanosheets. The 4T1 cells and L929 cells were seeded in 96‐well plates (1 × 10^4^ cells) for 12 h to attach. Then coincubation with W_1.33_C ‐BSA nanosheets at various concentrations (12–200 µg mL^−1^) for 24 h, the cell survival rate of 4T1 and L929 cells was tested using the typical CCK‐8 assay.

### In Vitro Photothermal Ablation Performance of W_1.33_C‐BSA Nanosheets

4T1 cells (10^4^ cells per well) were pre‐seeded in 96‐well plates and cultured overnight. Then, the culture medium was replaced by a fresh medium (100 µL) containing W_1.33_C‐BSA nanosheets at different amounts ( 12 to 200 µg mL^−1^) . After 4 h incubation, the cells were exposed to the 808 or 1064 nm laser at a power density of 1.25 W cm^−2^ for 10 min irradiation. After 2 h of treatment, the standard CCK‐8 assay was performed to access the cell viabilities.

### Investigation on Photothermal Ablation Performance of W_1.33_C‐BSA Nanosheets by Cell Apoptosis

4T1 cells were pre‐seeded in six‐well plates for 24 h. Then culture mediums containing different ingredients were added to the cell plates and incubated for another 4 h. The cells were disposed of as mentioned above and were collected by centrifugation. After washing with PBS, the cells were resuspended in 100 µL binding buffer and stained by Annexin V FITC and PI for 20 min. At last, flow cytometry was performed to observe cell apoptosis and necrosis.

### Intracellular Endocytosis as Observed by CLSM Observation

4T1 cells were incubated for 24 h in glass bottom culture dish (1 × 10^5^). Subsequently, the culture medium was replaced by an Roswell Park Memorial Institute （RPMI）‐1640 medium containing FITC‐labeled W_1.33_C‐BSA nanosheets (1 mL, 200 µg mL^−1^). Then the cells were incubated for 2, 4, and 8 h. After incubation , 100 µL 4',6‐diamidino‐2‐phenylindlole（DAPI） was supplemented for cell nuclei staining. After 15 min, the cells were then lightly washed with PBS and visualized by CLSM.

### In Vivo Photothermal Therapy

A 4T1 tumor‐bearing model was established by subcutaneous inoculation of 4T1 cells (1 × 10^6^) at the right back. Then the mice were randomized into six groups (*n* = 5), including control (G I), NIR I only (G II), NIR II only (G III), W_1.33_C‐BSA only (G IV), W_1.33_C‐BSA + NIR I (G V), and W_1.33_C‐BSA + NIR II (G VI). The dose of W_1.33_C‐BSA nanosheets was 20 mg kg^−1^. The mice were anesthetized 4 h after material injection and then subjected to laser irradiation. The experimental conditions were as follows: laser wavelength, 808/1064 nm; laser power density, 1.25 W cm^−2^ ; duration time, 5 min. The volume of tumors was measured every 2 d. In addition, temperature changes at the tumor site were recorded by IR thermal imaging camera (FLIRTM A325SC camera, USA). Meanwhile, CEUS was carried out on the GE Logiq S7 equipped with a linear array transducer of 3–5 MHz to assess to tumor elimination. Further, TUNEL staining, Ki‐67 antibody and CD31^+^ antibody were applied to observe tumor cell apoptosis, cell proliferation and vascular density, respectively.

### Statistical Analysis

All of the above quantitative data were shown as mean ± standard deviation (SD). Statistical analysis was carried out through Origin 2017 software. The results were considered to be statistically significant at *P < 0.05, **P < 0.01, and ***P < 0.001.

## Conflict of Interest

The authors declare no conflict of interest.

## Supporting information

Supporting Information

## Data Availability

Research data are not shared.
