# Supplementary material for: Biodegradable and Excretable 2D W1.33C i‐MXene with Vacancy Ordering for Theory‐Oriented Cancer Nanotheranostics in Near‐Infrared Biowindow
Source: Adv Sci (Weinh). 2021 Oct 29;8(24):2101043. doi: 10.1002/advs.202101043 (PMC8693041; doi:10.1002/advs.202101043)
Supplement: Supplementary file 1 — Supporting Information [file ADVS-8-2101043-s001.pdf]

© 2021 Wiley-VCH GmbH

ADVANCED SCIENCE
Open Access

Supporting Information

for *Adv. Sci.,* DOI: 10.1002/advs.202101043

Biodegradable and Excretable 2D W1.33C i-MXene with Vacancy Ordering for Theory-Oriented Cancer Nanotheranostics in Near Infrared Biowindow

Bangguo Zhou, Haohao Yin, Caihong Dong, Liping Sun, Wei Feng, Yinying Pu, Xiaoxia Han, Xiaolong Li, Dou Du, Huixiong Xu* and Yu Chen**

Supporting Information

Biodegradable and Excretable 2D $W_{1.33}C$ *i*-MXene with Vacancy Ordering for Theory-Oriented Cancer Nanotheranostics in Near Infrared Biowindow

Bangguo Zhou, Haohao Yin, Caihong Dong, Liping Sun, Wei Feng, Yinying Pu, Xiaoxia Han, Xiaolong Li, Dou Du, Huixiong Xu* and Yu Chen**

B. Zhou, H. Yin, Dr. L. Sun, Y. Pu, X. Han, X. Li, D. Du, Prof. H. Xu

Department of Medical Ultrasound, Shanghai Tenth People's Hospital, Ultrasound Research and Education Institute, Tongji University Cancer Center, Shanghai Engineering Research Center of Ultrasound Diagnosis and Treatment, Tongji University School of Medicine, Shanghai 200072, P. R. China. E-mail: xuhuixiong@tongji.edu.cn (H. Xu).

Dr. W. Feng, Prof. Y. Chen
School of Life Sciences, Shanghai University, Shanghai, 200444, P. R. China. Email: fengw@shu.edu.cn (W. Feng); chenyuedu@shu.edu.cn (Y. Chen).

Dr. C. Dong
Department of Ultrasound, Zhongshan Hospital, Fudan University, and Shanghai Institute of Medical Imaging, Shanghai, 200032, P. R. China.

A: Experimental Section

Materials and Reagents. Tungsten (W, 99.5%, Sigma-Aldrich), yttrium (Y, 99.5%, Sigma-Aldrich), Aluminum (Al, 99.5%, Sigma-Aldrich), carbon (C, 99.5%, Sigma-Aldrich) and bovine serum albumin (BSA, > 98%, Sigma-Aldrich) were obtained from Sigma-Aldrich (Shanghai) Trading Co., Ltd. (Shanghai, China). Lithium fluoride (LiF, 99.9% metal basis) was purchased from Aladdin Reagent Co., Ltd. (Shanghai, China). Hydrochloric acid (HCl) was obtained from Sinopharm Chemical Reagent Co., Ltd. (Shanghai, China). Calcein acetoxymethyl ester (calcein AM) and propidium iodide (PI) were both purchased from Shanghai Moyi Biotechnology Co., Ltd. (Shanghai, China). Acridine orange (AO) was obtained from Shanghai Bestbio Bioscience (Shanghai, China). Cell counting Kit-8 (CCK-8), 2-(4-Amidinophenyl)-6-indolecarbamidine dihydrochloride (DAPI) and annexin V-fluorescein isothiocyanate (FITC)/propidium iodide (PI) double staining kits were purchased from Beyotime Institute of Biotechnology (Haimen, Jiangsu, China). Fetal bovine serum (FBS), trypsin and penicillin/streptomycin were purchased from Gibco Life Technologies Co., Ltd. (Grand Island, USA). Dulbecco's modified Eagle medium (DMEM) and Roswell Park Memorial Institute (RPMI) 1640 medium were purchased from Hyclone Laboratories (Logan, Utah, USA). Paraformaldehyde was purchased from Beijing Dingguo Changsheng Biotechnology Co., Ltd. (Beijing, China). LysoTracker Green DND-26, Alexa Fluor 488 conjugated phalloidin, Triton X-100, MitoTracker Green FM and ER-Tracker™ Green were purchased from Thermo Fisher Scientific Inc. (Waltham, Massachusetts, USA). Deionized (DI) water was obtained from a water purification system (Milli-Q system, Millipore, USA).

Theoretical Calculations: All first-principles calculations based on density functional theory (DFT) were performed with the Vienna ab initio simulation package (VASP) using the projector augmented wave method with non-spinpolarized generalized gradient approximation (GGA) as parameterized by Perdew-Burke-Ernzerhof (PBE) for treating electron exchange and correlation effects. Wave functions were expanded in plane wavesup to an energy cutoff of 520 eV.

Characterization. The microstructure and composition were recorded by field emission scanning electron microscope (Magellan 400, FEI, USA) coupled with an energy dispersive spectrometer (EDS). Transmission electron microscopy (TEM) imaging was performed for the structure and morphology characterization of the synthesized samples using a JEM-2100F field-emission transmission electron microscope at an accelerating voltage of 200 kV (JEOL Company Ltd., Japan). Powder X-ray diffraction (XRD) pattern was carried out by using a Rigaku D/MAX-2200 PC XRD instrument (Rigaku Co. Ltd., Tokyo, Japan) equipped with Cu Kα radiation (λ = 1.54 Å) at 40 kV and 40 mA. Dynamic light scattering (DLS) and zeta potential measurements were measured on a Nano ZS 960 Malvern Zetasizer Nanoseries (Malvern Instrument Ltd. UK). UV−vis−NIR absorption spectra were conducted by a Shimadzu UV-3600 UV-vis-NIR scanning spectrometer (Shimadzu Scientific Instruments, Japan) with QS-grade quartz cuvettes of 1 cm optical path length at room temperature. The optical absorbance per cell length (A/L) was measured from the optical absorbance intensity at 808/1064 nm. The $W_{1.33}C$ extinction coefficient was extracted from the slope of a plot of A/L versus concentration from Beer's law (A/L = αC). The fluorescence spectra were conducted by Edinburch Instruments fluorescence spectrometers 980 (Edinburch Instruments Ltd.UK). The

$W_{1.33}C$ concentration was tested by an inductively coupled plasma optical emission spectrometry (ICP-OES) using an Agilent 725 ICP-OES System (Agilent Technologies, US). The confocal laser scanning microscopy (CLSM) images were obtained using FV1000 (Olympus Company, Japan).

***In Vitro* Photothermal Performance of $W_{1.33}C$ Nanosheets.** To evaluate photothermal performance of $W_{1.33}C$, the $W_{1.33}C$ nanosheets aqueous solution (100 μL) with different $W_{1.33}C$ concentrations (25, 50, 100 and 200 μg mL^{-1}) was measured and analyzed by continuously irradiating with an 808 nm or 1064 nm near-infrared (NIR) laser (Shanghai Connect Fiber Optics Company) at a power density of 1.25 W cm^{-2} for 5 min, respectively. The temperature at different time duration was continuously recorded by using a digital infrared (IR) thermal imaging equipment (FLIR A325SC camera, USA). Subsequently, the photothermal-stability performance of the $W_{1.33}C$ nanosheets was conducted. The $W_{1.33}C$ nanosheets dispersion (100 μL, 200 μg mL^{-1}) was exposed to 808 or 1064 nm laser at the power density of 1.25 W cm^{-2} for 5 min, and then cooling down by laser off for 10 min. The irradiation heating and natural cooling cycles were repeated for five times. The value of η was determined according to equation: $\eta = hS(T_{max} - T_{surr}) - Q_s / I(1-10^{-A_\lambda})$,Where h is the heat transfer coefficient, S represents the surface area of the sample container, T_{max} is the maximum equilibrium temperature, T_{surr} represents the temperature of the surrounding, respectively, Q_s is the heat energy dissipation associated with the light absorbed by the 96-well plate and solvent, I represents the NIR laser power density, and A_λ is the absorbance of the $W_{1.33}C$ at the wavelength (λ) of 808 or 1064 nm.

CLSM Observation of Photothermal-Ablation Effect of $W_{1.33}$C-BSA Nanosheets. 4T1 cells were cultured in glass bottom culture dish (35 mm × 10 mm) with a density of 1 × 10^5 and incubated for 24 h. After discarding the medium and washing with PBS, the cells were divided into six groups in above-mentioned manner, which were further incubated with calcein-AM (100 μL) and PI solution (100 μL) for 15 min. After that living cells and dead cells were then observed by CLSM.

CLSM Observation of Photothermal-Ablation Effect of $W_{1.33}$C-BSA Nanosheets on Cell Lysosome. For lysosome-damage assay, 4T1 cells were cultured into glass bottom culture dishes (35 mm × 10 mm) with a density of 1 × 10^5 and incubated for 24 h. After discarding the medium and washing with PBS, the cells were divided into the above-mentioned six groups, which were further incubated with LysoTracker Green DND-26 (200 μL, 50 × 10^{-9} M) staining solution at 37 °C for 15 min. After further PBS washing, the cells were visualized by confocal laser scanning microscope (CLSM).

CLSM Observation of Photothermal-Ablation Effect of $W_{1.33}$C-BSA Nanosheets on Cell Mitochondria. To explore the effect of photothermal ablation on cell mitochondria, 4T1 cells were cultured into glass bottom culture dishes (35 mm × 10 mm) with a density of 1 × 10^5 cells for 24 h. After discarding the medium and PBS washing, the cells were divided into the above-mentioned six groups, which were further incubated with MitoTracker Green FM (200 μL, 200 × 10^{-9} M) at 37 °C for 15 min in serum-free medium. After further PBS washing, the dishes were supplemented with serum-free medium, and then the cells were visualized by CLSM.

CLSM Observation of Photothermal-Ablation Effect of $W_{1.33}C$-BSA Nanosheets on Cell Cytoskeleton. To probe the effect of photothermal ablation on cell cytoskeleton, 4T1 cells were seeded into glass bottom culture dishes (35 mm × 10 mm) with the density of 1×10^5 cells and cultured for 24 h. Subsequently, the cells were divided into six groups into the above-mentioned six groups. After prewarmed PBS washing twice, 4T1 cells were treated with 4% paraformaldehyde for 30 min, and washed three times with PBS, then subjected to 0.1% TritonX-100 for 5 min, followed by blocking with 1% BSA for 30 min. After PBS washing, the F-actin of cells was stained by Alexa Fluor488 conjugated phalloidin at room temperature for 30 min. After washing with abundant PBS to remove residual dyes, the samples were visualized by CLSM.

CLSM Observation of Photothermal-Ablation Effect of $W_{1.33}C$-BSA Nanosheets on Cell Endoplasmic Reticulum (ER). To evaluate the effect of photothermal ablation on cell cytoskeleton, 4T1 cells were seeded into glass bottom culture dishes (35 mm × 10 mm) at a density of 1×10^5cells and cultured for 24 h. After PBS washing, the cells were divided into six groups in above-mentioned manner. After prewarmed PBS rinsing twice, 4T1 cells were stained with prewarmed ER-Tracker Green staining solution for 30 min at 37 °C. Subsequently the staining solution was replaced by fresh probe-free medium, those stained cells were viewed by CLSM.

***In Vivo* Biodistribution Study**. Biodistribution of $W_{1.33}C$-BSA in tumor and other main organs

was performed in 4T1 tumor-bearing mice (n = 3). 4T1 tumor-bearing mice were intravenously administered with $W_{1.33}C$-BSA (20 mg kg^{-1}) in PBS. Mice were dissected at predesignated time intervals (4, 24, and 48 h). Dissected organs were weighed, homogenized, and treated with strong acid. The $W_{1.33}C$-BSA distributions in different tissues were calculated as the percentage of injected dose per gram of tissue.

***In Vivo* Toxicity Study**. All animal experiments were in agreement with the guidelines of the Regional Ethics Committee for Animal Experiments and the care regulations approved by the administrative committee of laboratory animals of Shanghai Tenth People's Hospital. Healthy female Kunming mice (~ 18 g) were purchased and raised at Laboratory Animal Center, Shanghai Tenth People's Hospital. Kunming mice were randomly divided into 4 groups (n=5): (1) control group, (2) $W_{1.33}C$-BSA nanosheets in PBS (5 mg kg^{-1}), (3) $W_{1.33}C$-BSA nanosheets in PBS (10 mg kg^{-1}), (4) $W_{1.33}C$-BSA nanosheets in PBS (20 mg kg^{-1}). The body weight of mice was recorded every 2 days and scarified at the 30th day after intravenous administration of $W_{1.33}C$-BSA nanosheets. Serum biochemistry parameters including aspartate aminotransferase (AST), alanine aminotransferase (ALT), alkaline phosphatase (ALP), creatinine (Crea) and urea of blood supernatant (harvest by centrifugation) were recorded using Beckman Coulter Unicel DxC 800 automatic biochemical analyzer. Routine blood test including white blood cell (WBC), red blood cell (RBC), hemoglobin (HGB), hematocrit (HCT), mean corpuscular hemoglobin (MCH), mean corpuscular hemoglobin concentration (MCHC) and platelet count (PLT) were measured on Sysmex XS-800i automated hematology analyzer. Then, the major organs (heart, liver, spleen, lung, and kidney) were sectioned into slices and stained with hematoxylin-eosin

staining (H&E) for histological analysis.

***In Vitro* and *In Vivo* Computed Tomography Imaging.** For *in vitro* computed tomography (CT) imaging, to evaluate the CT imaging performance of $W_{1.33}C$-BSA nanosheets, different concentrations of $W_{1.33}C$-BSA nanosheets (0.1, 0.3, 0.6, 1.3, 2.5, 5 and 10 mg mL^{-1}) dissolved in xanthan gum solution were used for *in vitro* CT imaging to obtain a linear curve between $W_{1.33}C$-BSA nanosheet concentration and CT signal. For *in vivo* CT imaging, $W_{1.33}C$-BSA nanosheets (20 mg kg^{-1}) were intravenously administrated into 4T1 tumor-bearing nude mice. CT imaging of tumor-bearing mice were recorded before and after the intravenous administration of $W_{1.33}C$-BSA nanosheets. The *in vitro* and *in vivo* CT imaging were performed on GE discovery CT750 HD (GE Healthcare). The parameters were as follows: beam pitch = 0.531:1, slice thickness = 0.625 mm, gantry rotation time = 0.5 s and speed = 10.62 mm.

***In Vitro* and *In Vivo* Photoacoustic Imaging.** For *in vitro* PA imaging, to assess the linearity of the PA signals as a function of $W_{1.33}C$-BSA nanosheets concentration, different concentrations of $W_{1.33}C$ nanosheets (0.06, 0.12, 0.25, 0.5 and 1 mg mL^{-1}) dispersed in deionized water were used for PA signal test. For *in vivo* PA imaging, $W_{1.33}C$ nanosheets (20 mg kg^{-1}) were intravenously injected into 4T1 tumor-bearing mice.PA imaging of 4T1 tumor-bearing mice were imaged before and after the injection of $W_{1.33}C$-BSA nanosheets. Vevo LAZR PA Imaging System was selected to take *in vitro* and *in vivo* PA imaging experiment.

Transcriptomic and proteomics analysis. Mice were randomly divided into 2 groups (n = 3 per

group), including (1) control group and (2) treat group (intravenous injection of $W_{1.33}C$-BSA nanosheet solution followed by irradiation of the NIR II (1064 nm) laser at a power density of 1.25 W cm^{-2} for 5 min). To prevent protein and RNA degradation, tumor tissues were collected for sequencing at 4 hours after treatment. The tissues were subsequently sequenced and analyzed.

B: Supplementary figures

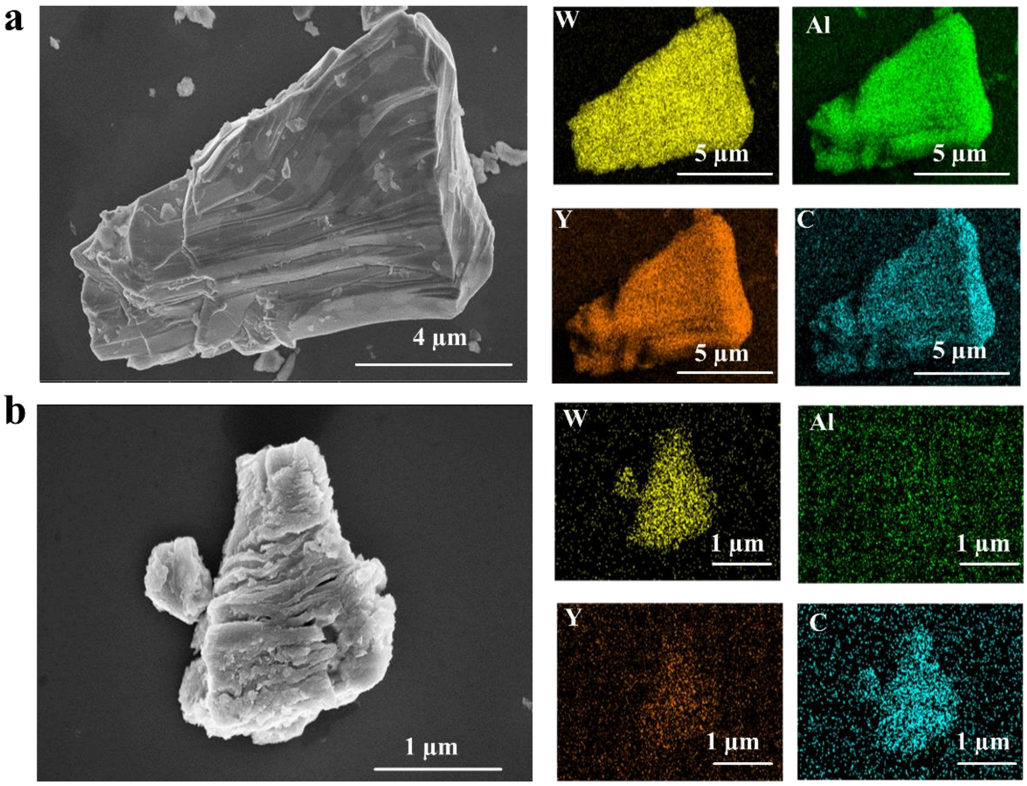

Figure S1. SEM and the corresponding elemental-mapping images (W, Y, Al and C elements) of $(W_{2/3}Y_{1/3})_2AlC$ *i*-MAX (a) before etching and (b) after LiF/HCl etching.

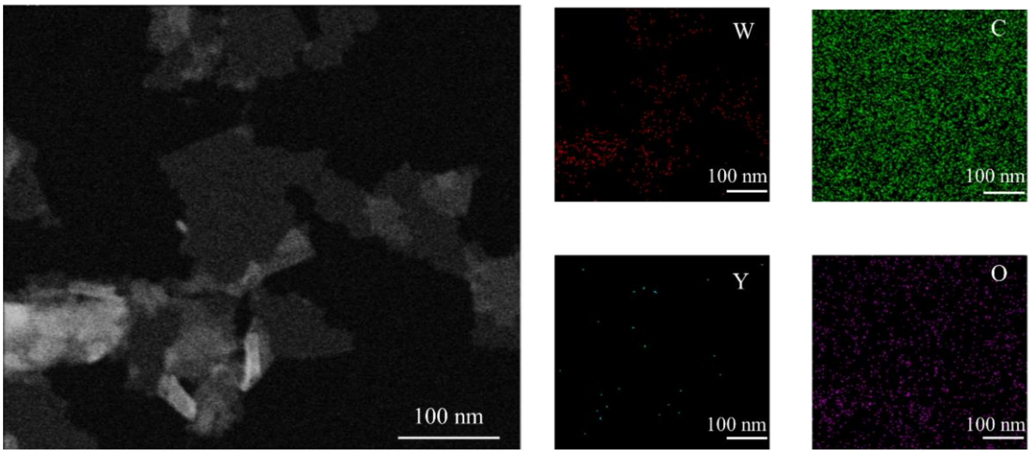

Figure S2. Dark-field TEM images of $W_{1.33}C$ nanosheets and corresponding EDS elemental mapping.

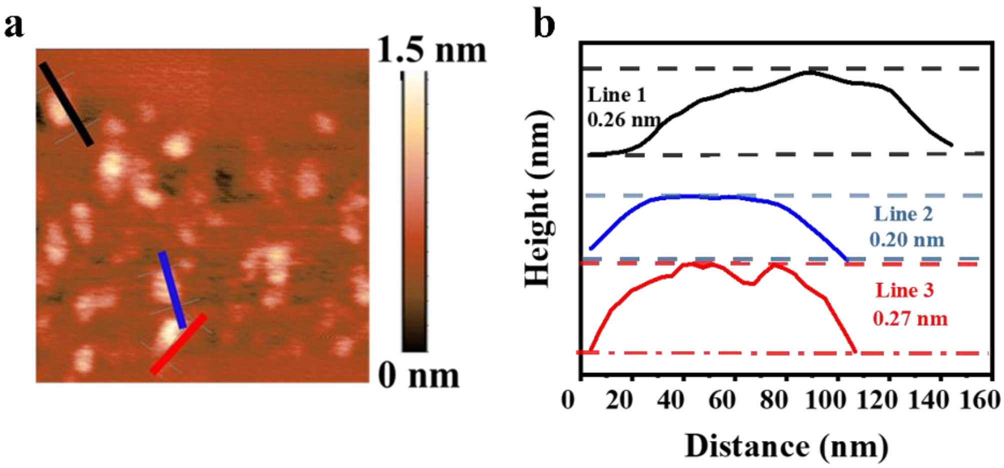

Figure S3. (a) Typical AFM image and (b) thickness analysis of $W_{1.33}C$ *i*-MXene.

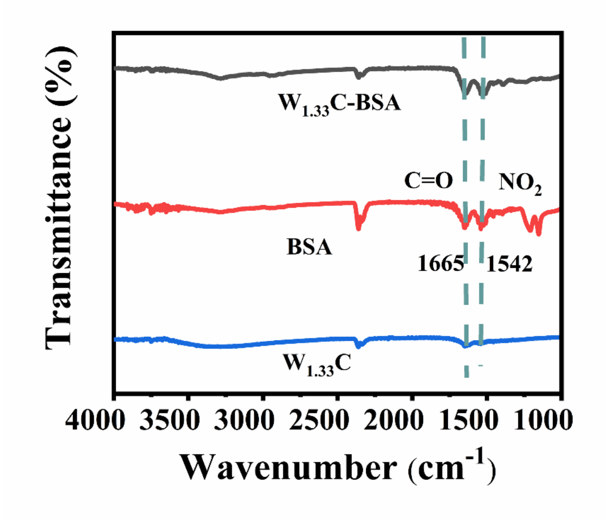

Figure S4. FTIR patterns of $W_{1.33}C$-BSA nanosheets, BSA and $W_{1.33}C$ *i*-MXene.

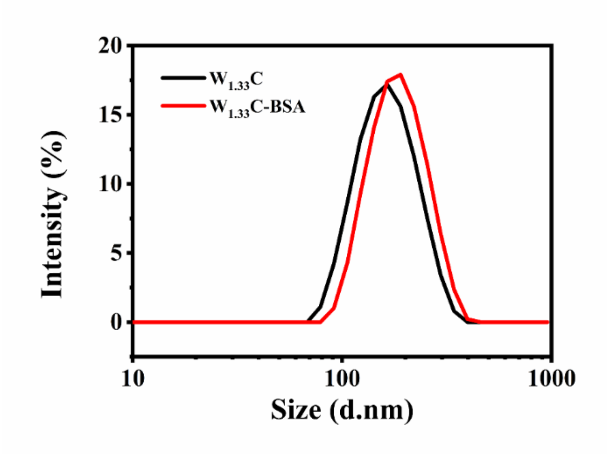

Figure S5. DLS analysis of $W_{1.33}C$ *i*-MXene and $W_{1.33}C$-BSA nanosheets.

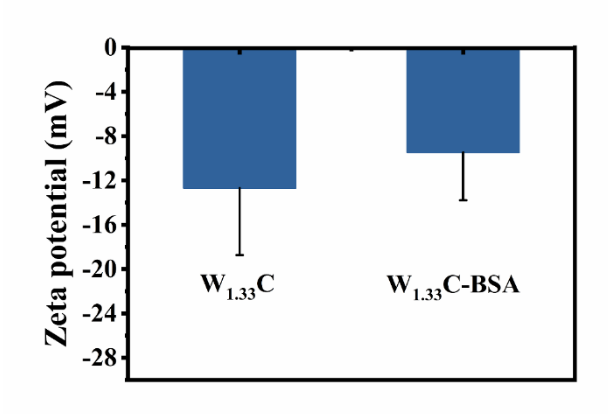

Figure S6. Zeta potential analysis of $W_{1.33}C$ *i*-MXene and $W_{1.33}C$-BSA nanosheets.

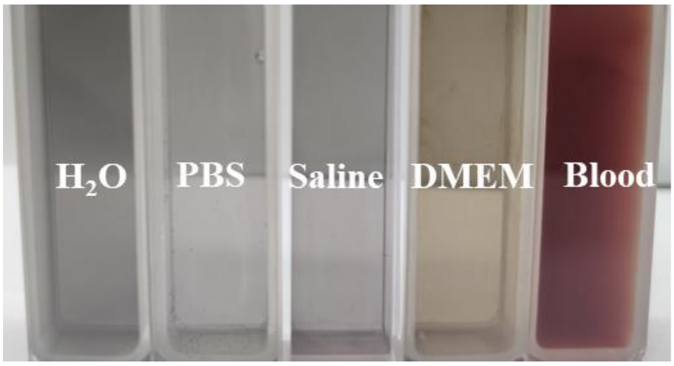

Figure S7. Photographs of $W_{1.33}C$-BSA nanosheets dispersed in various solvents.

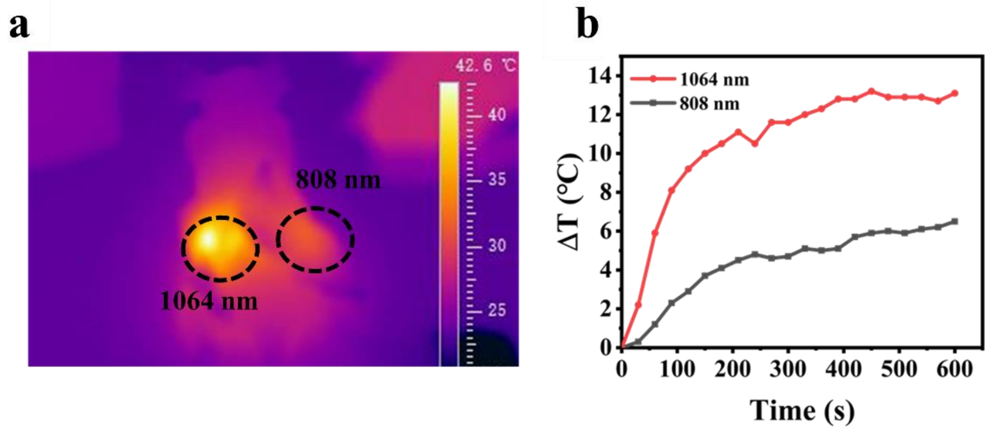

Figure S8. IR thermal images and the corresponding temperature changes of 4T1-tumor-bearing mice injected with $W_{1.33}C$-BSA nanosheets under both 808 and 1064 nm lasers irradiation.

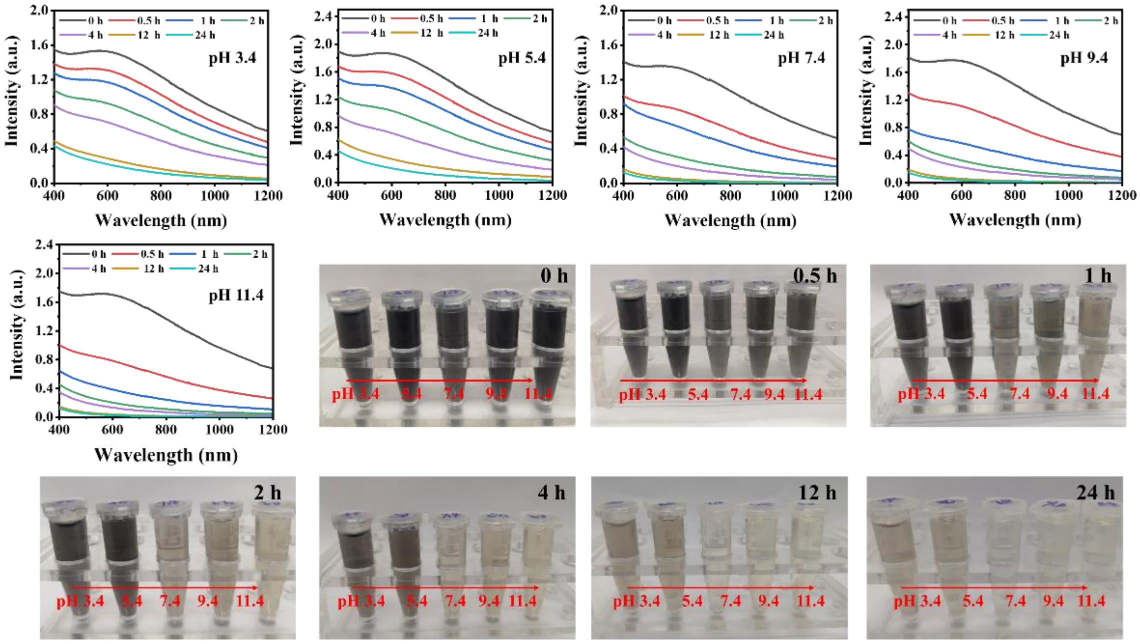

Figure S9. Degradation process of $W_{1.33}C$-BSA nanosheets dispersed in PBS with different pH ranging from 3.4 to 11.4, as observed by UV–vis–NIR absorption spectra and corresponding digital photographs at a series of designed time points.

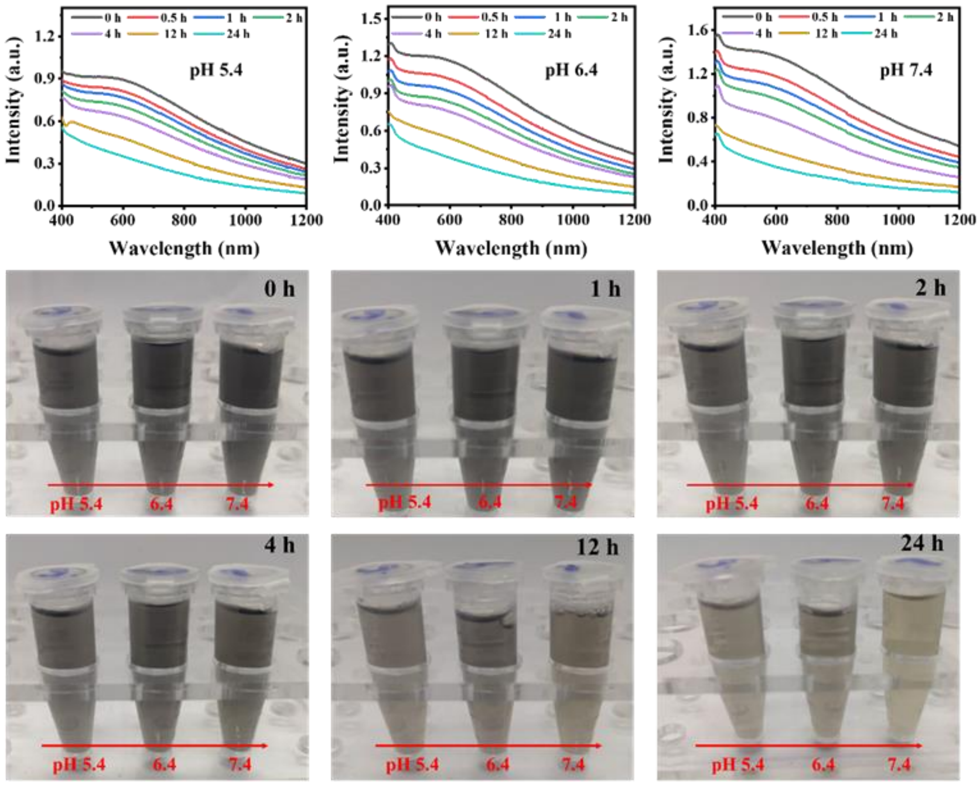

Figure S10. Degradation process of $W_{1.33}C$-BSA nanosheets in various pH values of RPMI 1640 medium containing 10% FBS (pH of 5.4, 6.4 and 7.4), as observed by absorption spectra and corresponding digital photographs at designed time points.

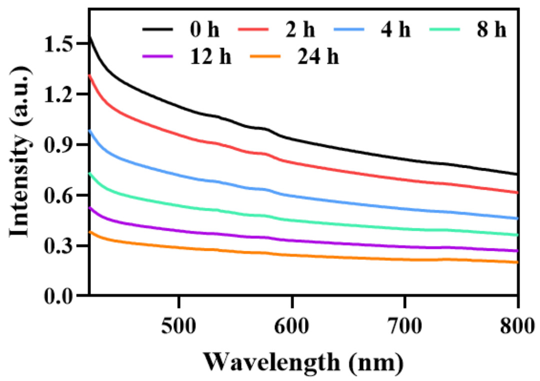

Figure S11. Degradation process of $W_{1.33}C$-BSA nanosheets in PBS containing 10% mouse blood as observed by absorption spectra.

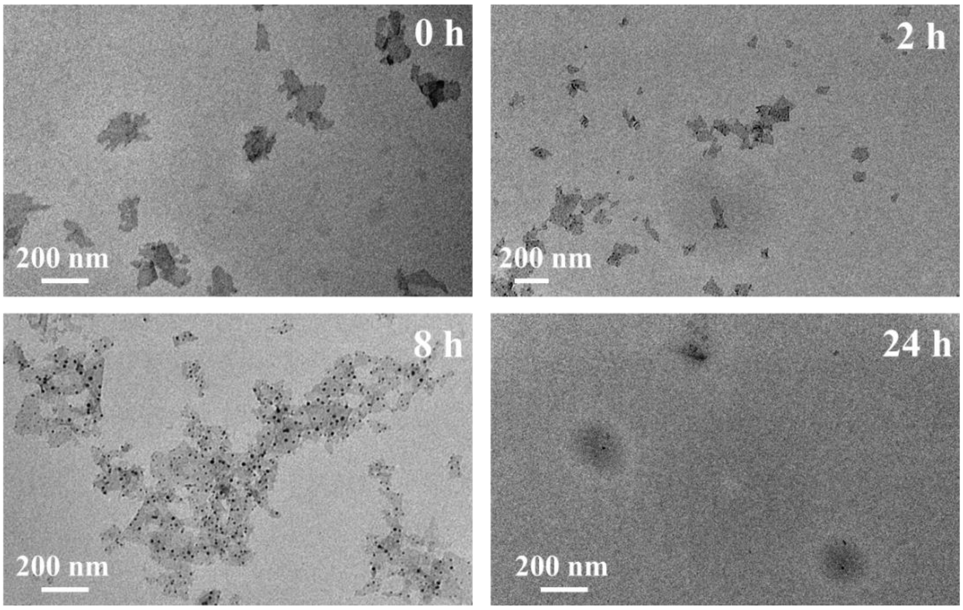

Figure S12.TEM images of the degradation product of $W_{1.33}C$-BSA nanosheets incubated in PBS (pH 7.4) at various time points.

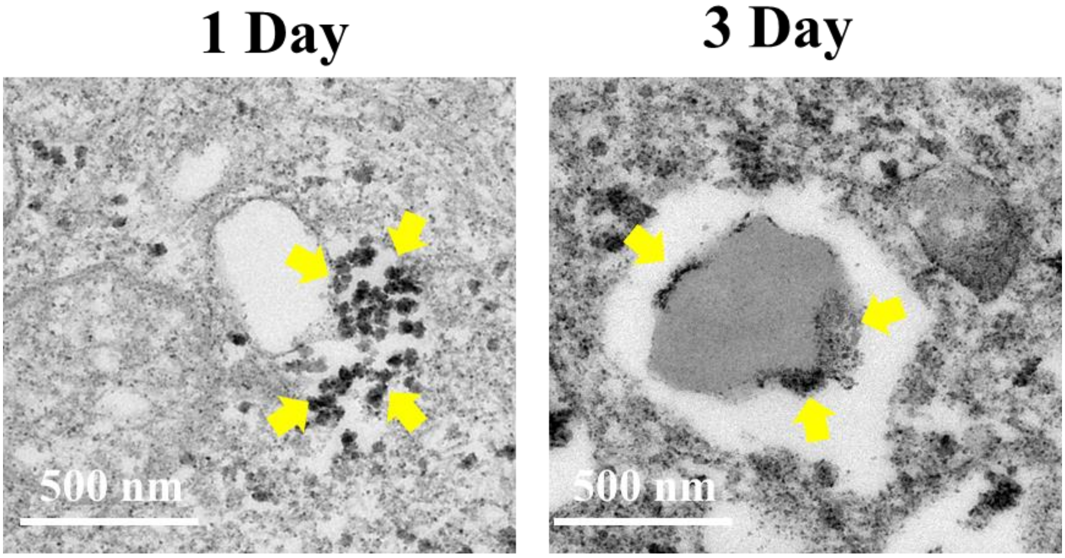

Figure S13. Intracellular biodegradation behavior and structural evolution of $W_{1.33}C$-BSA in 4T1 cells by bio-TEM observation after different incubation durations.

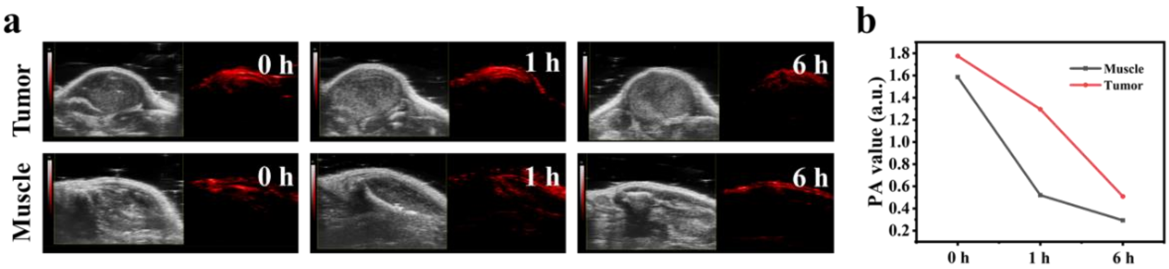

Figure S14. (a) Ultrasound and PA imaging of the tumor and muscle at 0, 1 and 6 h post injection of $W_{1.33}C$-BSA nanosheets. (b) Relative PA signal intensities at 0, 1 and 6 h post injection in the tumor and muscle region.

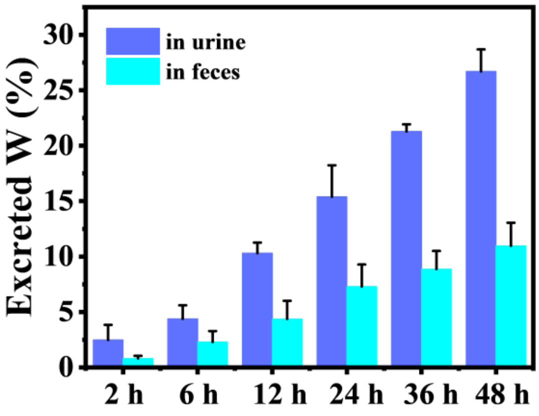

Figure S15. Cumulative excretion of W (in feces and urine) out of the mice (*n* = 3) after injection of $W_{1.33}C$-BSA nanosheets for predesigned time intervals (2, 6, 12, 24, 36 and 48 h).

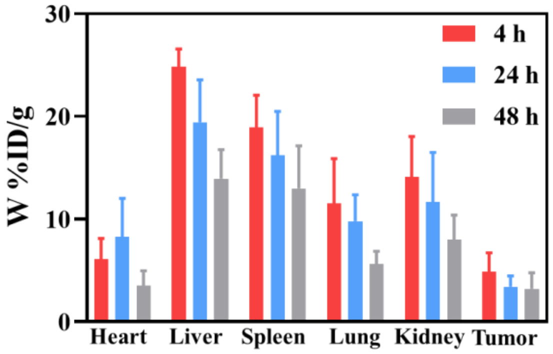

Figure S16. Biodistribution of W (% ID of W per gram of tissues) in main tissues and tumor after intravenous administration of $W_{1.33}C$-BSA nanosheets dispersed in PBS for varied time intervals (4, 24 and 48 h) (n = 3).

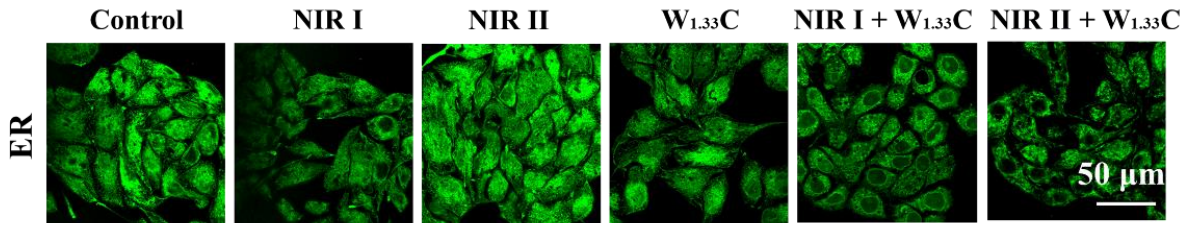

Figure S17. CLSM fluorescence images of 4T1 cells stained by ER-Tracker (green for endoplasmic reticulum).

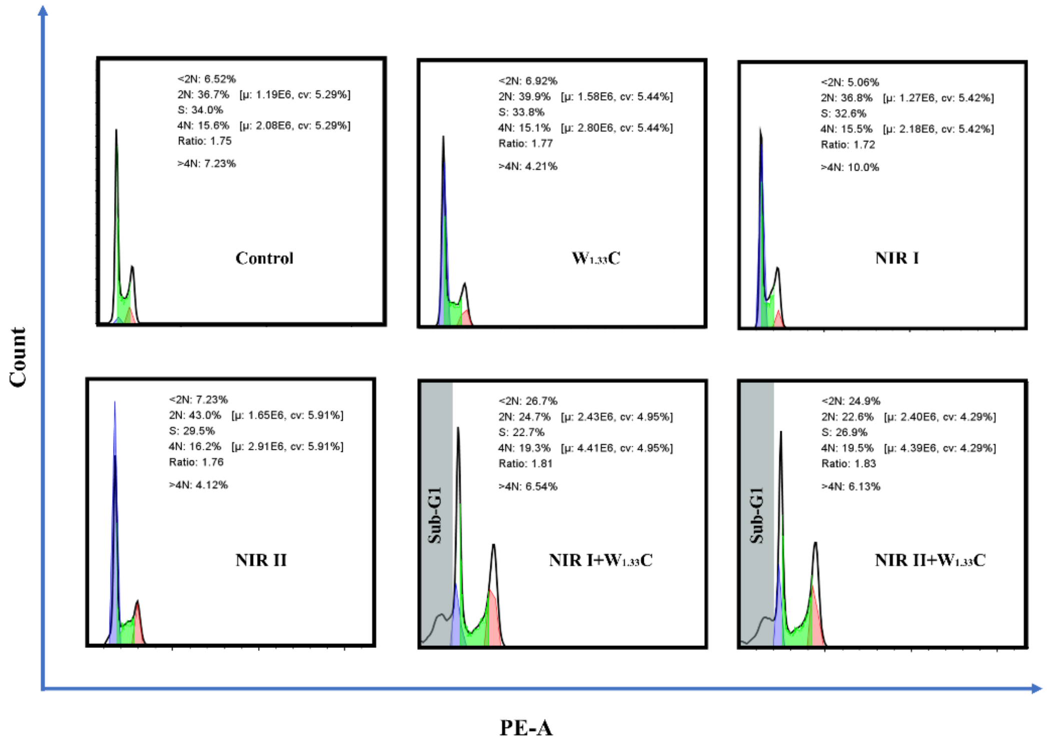

Figure S18. Histograms of 4T1 cells cycle distribution after various treatments.

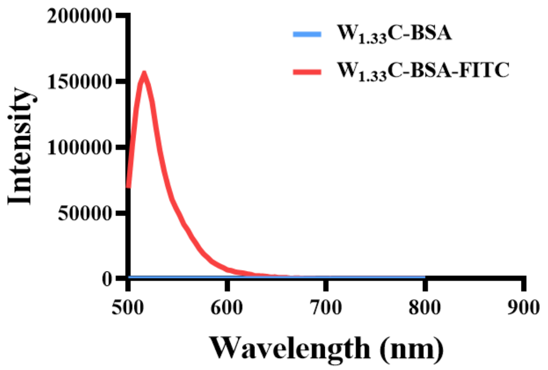

Figure S19. Fluorescence analysis of $W_{1.33}C$-BSA nanosheets and $W_{1.33}C$-BSA-FITC nanosheets.

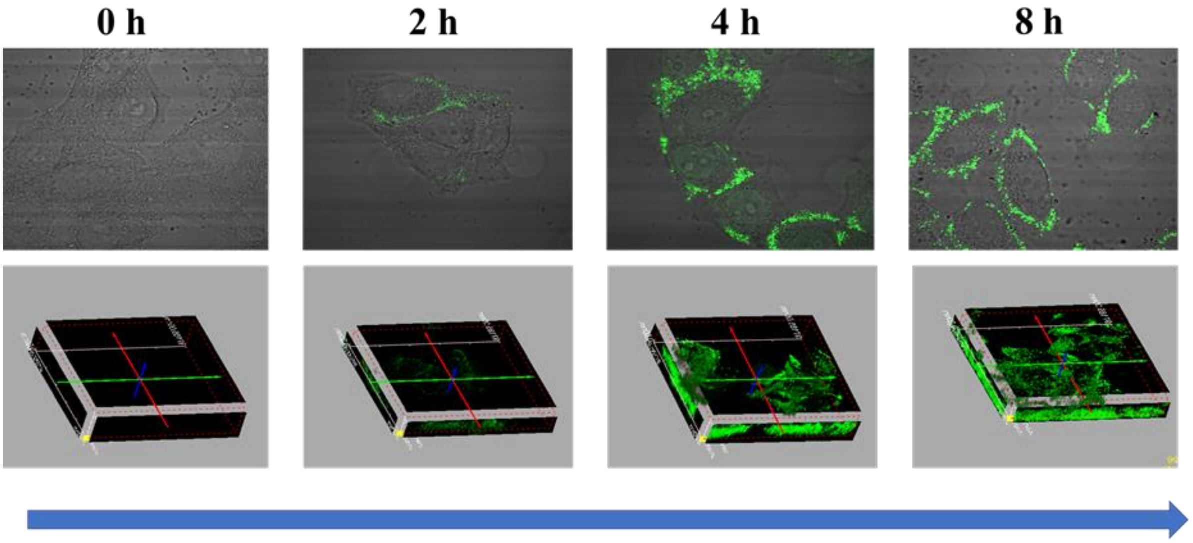

Figure S20. CLSM images of 4T1 cells incubated with FITC-labeled $W_{1.33}C$-BSA nanosheets for 0, 2, 4 and 8 h.

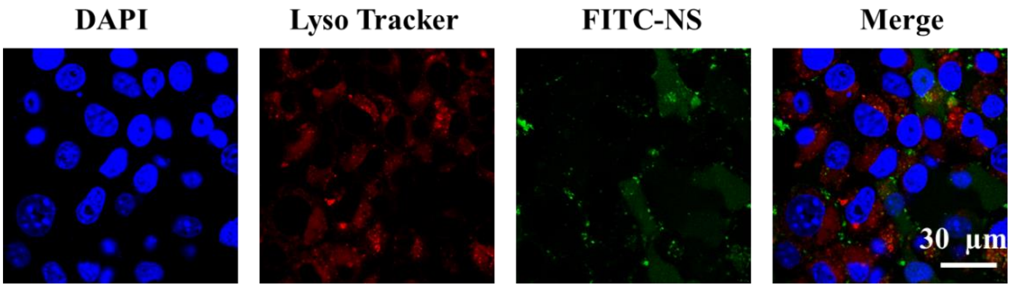

Figure S21. Typical confocal images of 4T1 cancer cells incubated with $W_{1.33}C$-BSA nanosheets. The cell nuclei were stained using DAPI (blue), the lysosomes were stained using LysoTracker Red (red), and $W_{1.33}C$-BSA nanosheets were labelled with FITC (green).

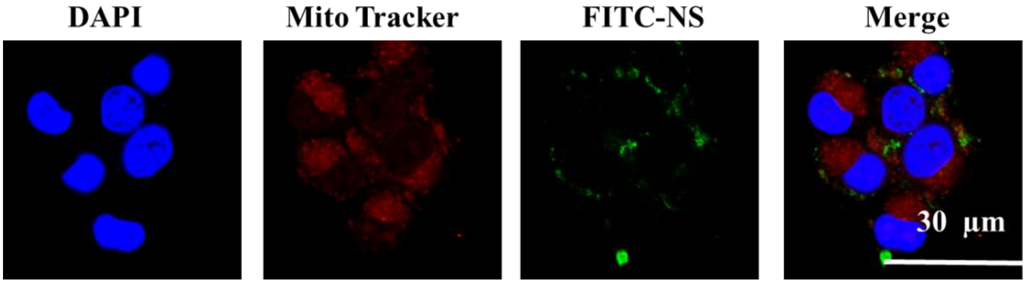

Figure S22. Typical confocal images of 4T1 cancer cells incubated with $W_{1.33}C$-BSA nanosheets. The cell nuclei were stained using DAPI (blue), the mitochondria were stained using MitoTracker Red (red), and $W_{1.33}C$-BSA nanosheets were labelled with FITC (green).

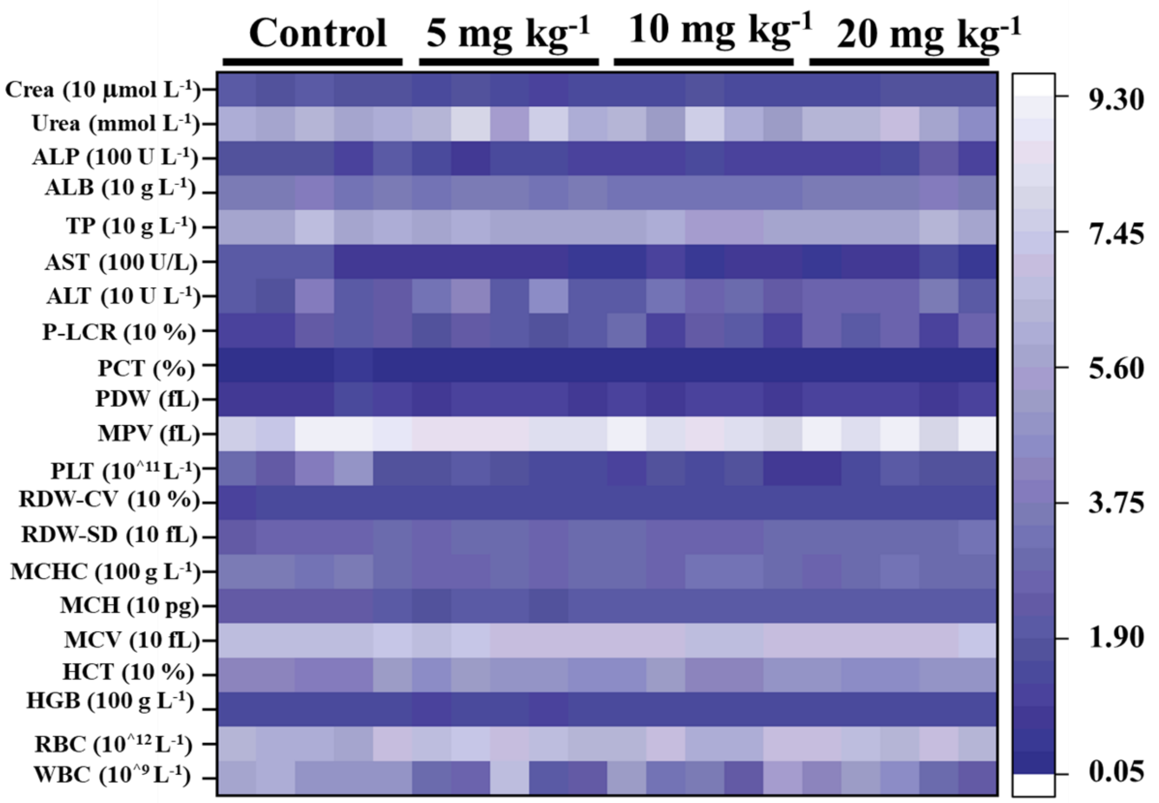

Figure S23. Blood routine and biochemistry indexes of Kunming mice in 28 d postinjection after different treatments.

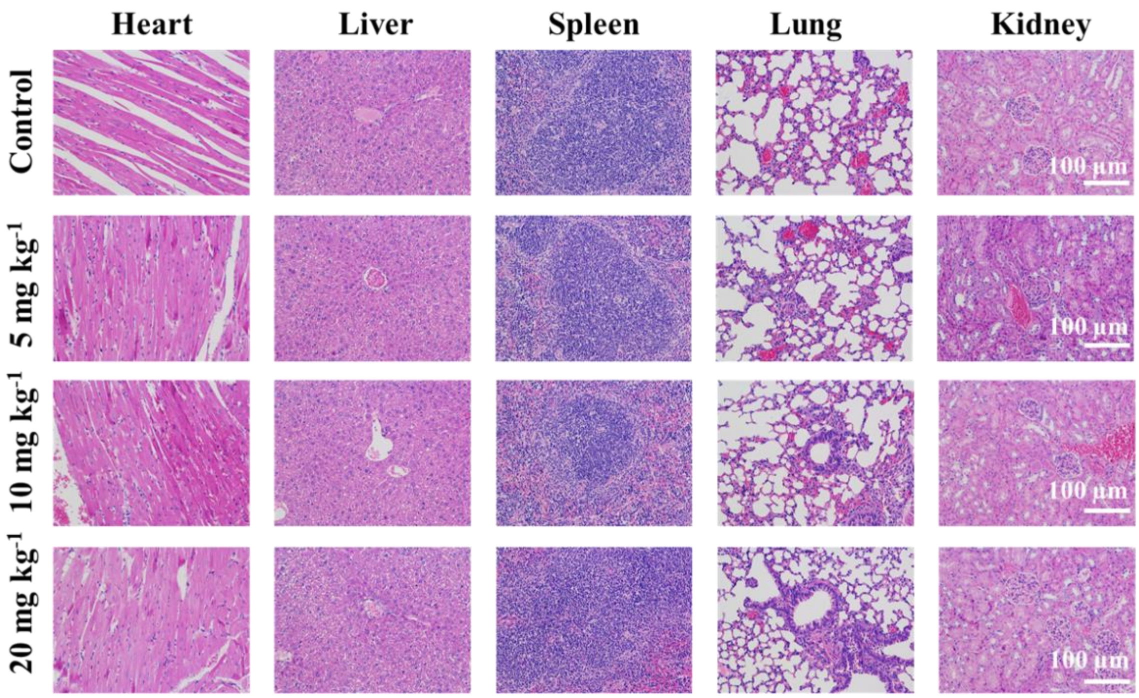

Figure S24. H&E stained microimages of heart, liver, spleen, lung and kidney at the 28th day from different treatment groups.

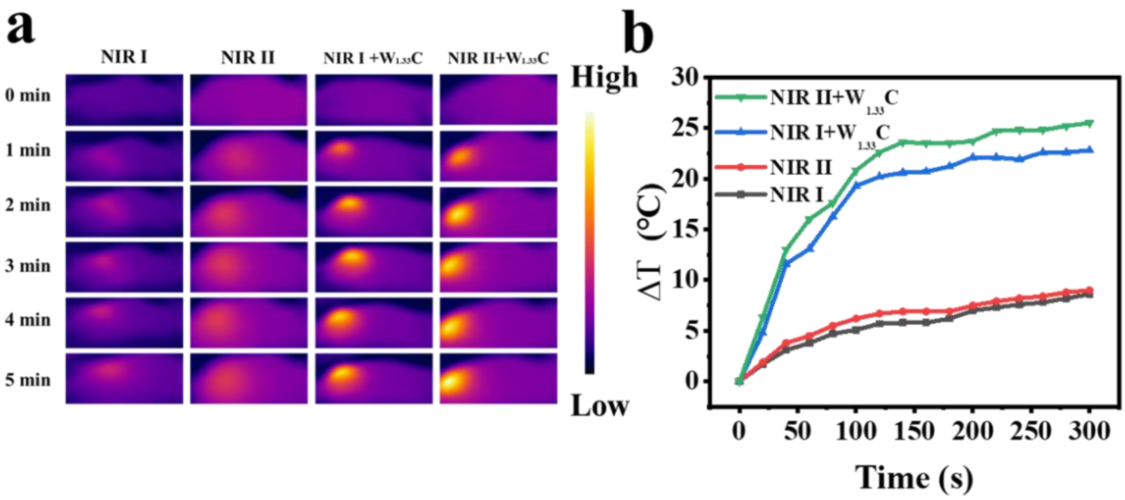

Figure S25. (a) The IR thermal images and (b) the corresponding temperature elevations at the tumor site of 4T1 tumor-bearing mice in different treatment groups during laser irradiation.

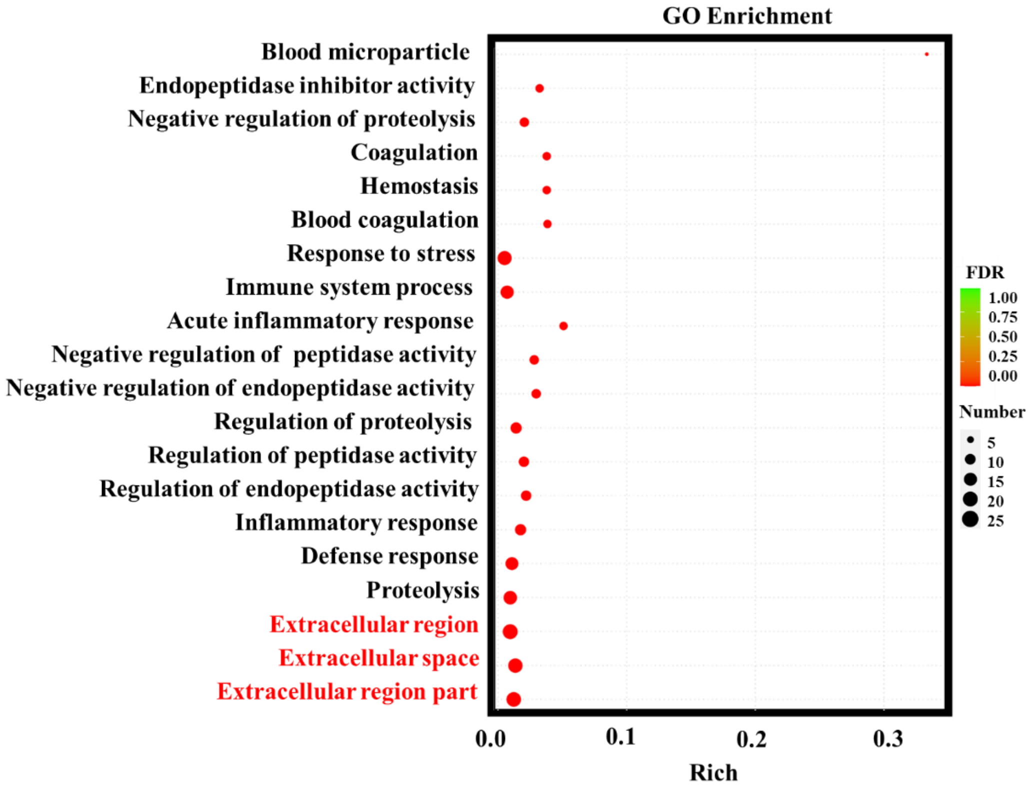

Figure S26. GO classification (biological process, molecular function and cellular component) of all differential proteins and enriched GO terms in top 20.
